# Extracellular Vesicle‐Based miRNA Profiling in Metabolic Dysfunction‐Associated Steatotic Liver Disease: A Secondary Analysis of the MULTISITE Clinical Trial

**DOI:** 10.1002/jex2.70173

**Published:** 2026-08-18

**Authors:** Katja Lund Cliff, Dominic Guanzon, Andrew Lai, Katherin Scholz‐Romero, Gunna Christiansen, Aase Handberg, Carlos Salomon, Maiken Mellergaard

**Affiliations:** ^1^ Department of Clinical Biochemistry Aalborg University Hospital Aalborg Denmark; ^2^ Department of Clinical Medicine Aalborg University Aalborg Denmark; ^3^ Translational Extracellular Vesicles in Obstetrics and Gynae‐Oncology Group, Frazer Institute, Faculty of Health, Medicine and Behavioural Sciences The University of Queensland Brisbane Australia; ^4^ UQ Centre for Extracellular Vesicle Nanomedicine (A.N.D., S.N., C.S.) The University of Queensland Brisbane Australia; ^5^ Department of Health Science and Technology Aalborg University Aalborg Denmark

**Keywords:** biomarkers, EVs, MASLD, miRNA, NAFLD, obesity

## Abstract

Metabolic dysfunction‐associated steatotic liver disease (MASLD) is the worldwide leading cause of liver‐related mortality with a prevalence of 75% among individuals with obesity and lacking screenings tools. Extracellular vesicles (EVs)‐based microRNAs (miRNAs) have emerged as promising biomarkers with important roles in the pathogenesis of MASLD. This study aimed to characterize EV‐based miRNA profiles in individuals with obesity and MASLD before and during weight loss intervention. Small RNA sequencing was used to profile EV‐miRNAs from plasma across three groups: individuals with obesity and MASLD (*n* = 35), individuals with obesity without hepatic steatosis (*n* = 24) and lean controls without hepatic steatosis (*n* = 26). Further, the MASLD group underwent a personalized weight‐loss intervention. The liver and MASLD‐related miR‐122‐5p and three other miRNAs were differentially expressed in the MASLD group compared with the obesity control group at baseline. Moreover, miR‐122‐5p correlated with liver fat and liver enzymes (ALT, AST and GGT) at baseline, revealed solid predictability for MASLD in a combined panel (AUC = 0.8), and lastly demonstrated the highest accuracy in identifying individuals with high liver fat among all individuals with obesity. Finally, several MASLD‐ and hepatocellular carcinoma‐related miRNAs decreased significantly following weight loss and liver fat reduction in the MASLD group.

## Graphical abstract

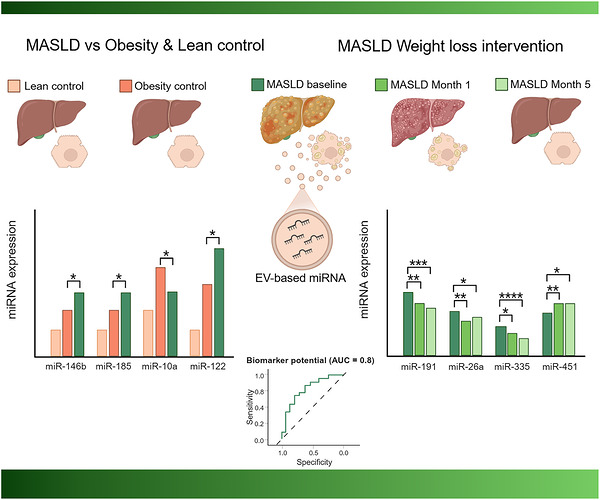

**Abbreviations**
ALT: Alanine aminotransferase
AST: Aspartate aminotransferase
BH: Benjamini‐Hochberg
BMI: Body mass index
CVD: Cardiovascular disease
EVs: Extracellular vesicles
FDR: False discovery rate
FC: Fold change
GGT: Gamma‐glutamyl transferase
GO: Gene Ontology
GAI: Generative artificial intelligence
HCC: Hepatocellular carcinoma
HDL: High‐density lipoprotein
HOMA‐IR: Homeostatic model assessment of insulin resistance
IEM: Immuno electron microscopy
IR: Insulin resistance
KEGG: Kyoto Encylopedia of Genes and Genomes
LOOCV: Leave‐one‐out cross‐validation
LDL: Low‐density lipoprotein
MRI: Magnetic resonance imaging
MASLD: Metabolic dysfunction‐associated steatotic disease
MASH: Metabolic dysfunction‐associated steatohepatitis
Mets: Metabolic syndrome
miRNA: MicroRNA
MW: Molecular Weight
NTA: Nanoparticles tracking analysis
NPV: Negative predictive value
NGS: Next generation sequencing
NAFLD: Non‐alcoholic fatty liver disease
PCR: Polymerase chain reaction
PPV: Positive predictive value
PDFF: Proton density fat fraction
RIPA: Radioimmunoprecipitation assay buffer
ROC: Receiver Operating Characteristic
rpm: Revolutions per minute
RT: Room temperature
SIRT1: Sirturin‐1
TG: Triglycerides
T2D: Type 2 diabetes
VST: Variance Stabilizing Transformation

## 1 Introduction

The worldwide prevalence of obesity has doubled since 1980, affecting nearly one‐third of the World´s population (Chooi et al. 2019). Obesity is the main risk factor for metabolic dysfunction‐associated steatotic liver disease (MASLD) which has a prevalence of 75% in individuals with obesity (Li et al. 2024; Younossi et al. 2024). MASLD, formerly known as NAFLD (defined as liver steatosis (>5% ectopic liver fat) in the absence of alcohol abuse) (Byrne and Targher 2024), was suggested as a nomenclature change in 2023, in part due to its strong association with cardiometabolic risk factors (Rinella et al. 2023). MASLD is defined as liver steatosis and at least one of the five cardiometabolic risk factors (Rinella et al. 2023; Chan et al. 2023). MASLD can progress to metabolic dysfunction‐associated steatohepatitis (MASH), liver fibrosis and cirrhosis and associate with an increased risk of developing type 2 diabetes (T2D), cardiovascular disease (CVD) and hepatocellular carcinoma (HCC) (Li et al. 2024; Chan et al. 2023). At present, diagnosis of liver steatosis is limited to liver biopsy or MRI, both unfeasible for screening purposes due to the invasive and high‐risk nature or being too costly and time consuming, respectively (Li et al. 2024; Chan et al. 2023; Garcia et al. 2023).

MicroRNA (miRNA) are small (21–24 nucleotides) non‐coding RNAs capable of regulating gene expression at the post‐transcriptional level (Tobaruela‐Resola et al. 2025). miRNAs can modify multiple biological functions related to the pathogenesis of MASLD, including lipid and glucose metabolism, triglyceride accumulation and metabolic stress response (Tobaruela‐Resola et al. 2025; Ezaz et al. 2020). Several circulating miRNAs have been associated with NAFLD and MASLD, including miRNA‐122, miRNA‐34a and miRNA‐21 (Tobaruela‐Resola et al. 2025; Carpi et al. 2024; Hochreuter et al. 2022). Nonetheless, circulating miRNAs analysis may be impacted by components like proteins, lipids and red blood cells (Ban and Song 2022). On the other hand, miRNAs derived from extracellular vesicles (EVs) are considered more stable and enriched, thus making them more suitable biomarker candidates (Cheng et al. 2014; Caviglia et al. 2025). EVs are heterogeneous membrane‐enclosed nanosized vesicles that play an important role in cell‐to‐cell communication by inducing signal transduction through the delivery of their cargo, such as miRNA, DNA, proteins and lipids (Jiang et al. 2023; Welsh et al. 2024). Furthermore, EVs can target specific cells and carry surface molecules specific to their cell or origin (Jiang et al. 2023). We recently showed that liver‐derived EVs and CD36‐expressing EVs were increased in individuals with obesity and MASLD and decreased during weight loss (Mellergaard et al. 2026). Moreover, EVs play key roles in inducing apoptosis in hepatocytes, and the pathogenesis and progression of MASLD (Jiang et al. 2023). In the present study, we hypothesized that specific EV‐based miRNA profiles differ between individuals with obesity and MASLD compared with an obesity control group, and that changes occur in the MASLD group during weight loss intervention. Thus, the aim of this study was to identify EV‐based miRNA profiles as potential non‐invasive biomarkers for early MASLD diagnosis among individuals with obesity.

## 2 Materials and Methods

### 2.1 Study Population and Design

**FIGURE 1:**
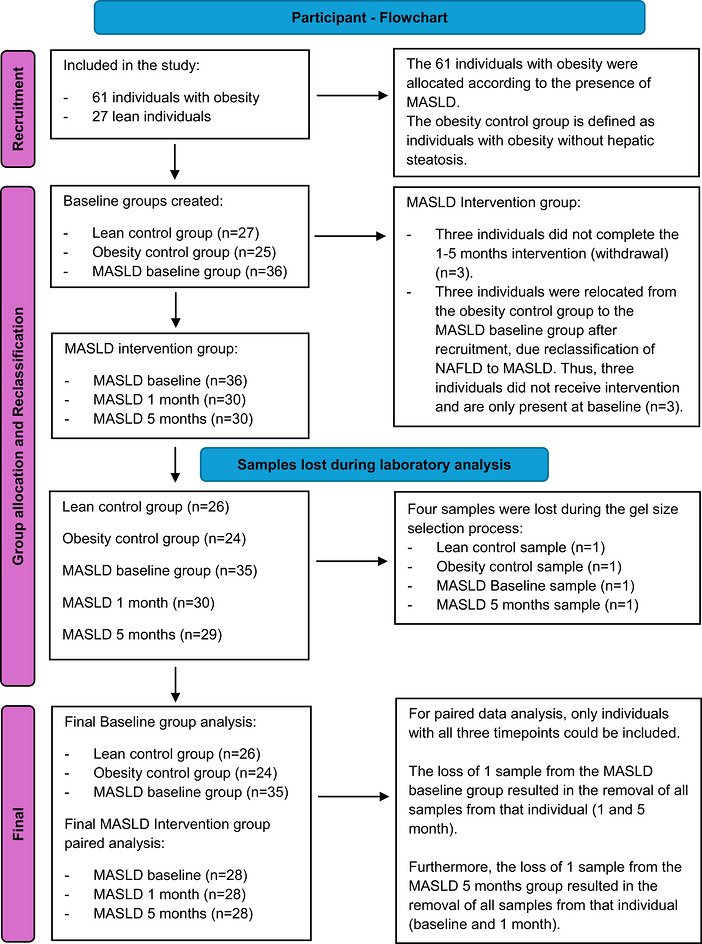
Participant flowchart. Participant flowchart showing the recruitment, initial group allocation, reclassification resulting from the change in nomenclature from NAFLD to MASLD, sample loss, and the final sample size included in data analysis.

This study is a secondary analysis of the MULTISITE study, registered on ClinicalTrials.gov (NCT05699863), conducted in accordance with the Helsinki Declaration and approved by the Health Ethics Committee of North Jutland, Denmark (N‐20200013). This case‐control study was completed at Aalborg University Hospital (Denmark), and involved a longitudinal personalized weight loss intervention of MASLD which was conducted by “Diætisthuset Aalborg” (Denmark) as previously described (Askeland et al. 2025). In brief, 61 non‐diabetic individuals with obesity (Body mass index [BMI]: 30.0–39.9 kg/m^2^) and 27 lean healthy individuals (BMI: 18.5–24.9 kg/m^2^), 30–60 years were included (Figure 1). Individuals with obesity were allocated according to presence of MASLD (Rinella et al. 2023; Chan et al. 2023), resulting in three baseline groups: individuals with obesity and MASLD (MASLD group, *n* = 36), individuals with obesity without hepatic steatosis (Obesity control group, *n* = 25), and lean individuals without hepatic steatosis (Lean control group, *n* = 27). Furthermore, the individuals in the MASLD group underwent a weight loss intervention with personalized diet and exercise advice for 5‐months.

### 2.2 Sample Collection

Fasting blood plasma samples, clinical data (anthropometry and blood pressure) and MRI were collected and performed at baseline for all participants and after 1‐ and 5‐months of weight loss intervention for the MASLD group. EDTA stabilized blood samples were centrifuged at 2 × 2500 *g* for 15 min to obtain platelet‐poor plasma and stored at −80°C. Liver steatosis was assessed by magnetic resonance imaging (MRI) using proton density fat fraction (PDFF), and alanine aminotransferase (ALT), aspartate aminotransferase (AST), gamma‐glutamyl transferase (GGT), cholesterol, high‐density lipoprotein (HDL), triglycerides (TG), low‐density lipoprotein (LDL), homeostatic model assessment of insulin resistance (HOMA‐IR), insulin and anthropometrics were measured as previously described (Askeland et al. 2025).

### 2.3 Group Allocation and Reclassification

Among the 36 individuals in the MASLD baseline group, only 30 completed the intervention and sampling at all three timepoints due to withdrawal (*n* = 3) and MASLD reclassification (*n* = 3) (Figure 1). To clarify, this study was performed prior to the reclassification of NAFLD to MASLD, and the intervention group was registered as individuals with obesity, NAFLD and the metabolic syndrome (MetS), as previously described (Mellergaard et al. 2026). Applying the current MASLD definition, three individuals from the original obesity control group were regrouped to the MASLD baseline group after the study was completed and therefore did not undergo intervention.

**FIGURE 2:**
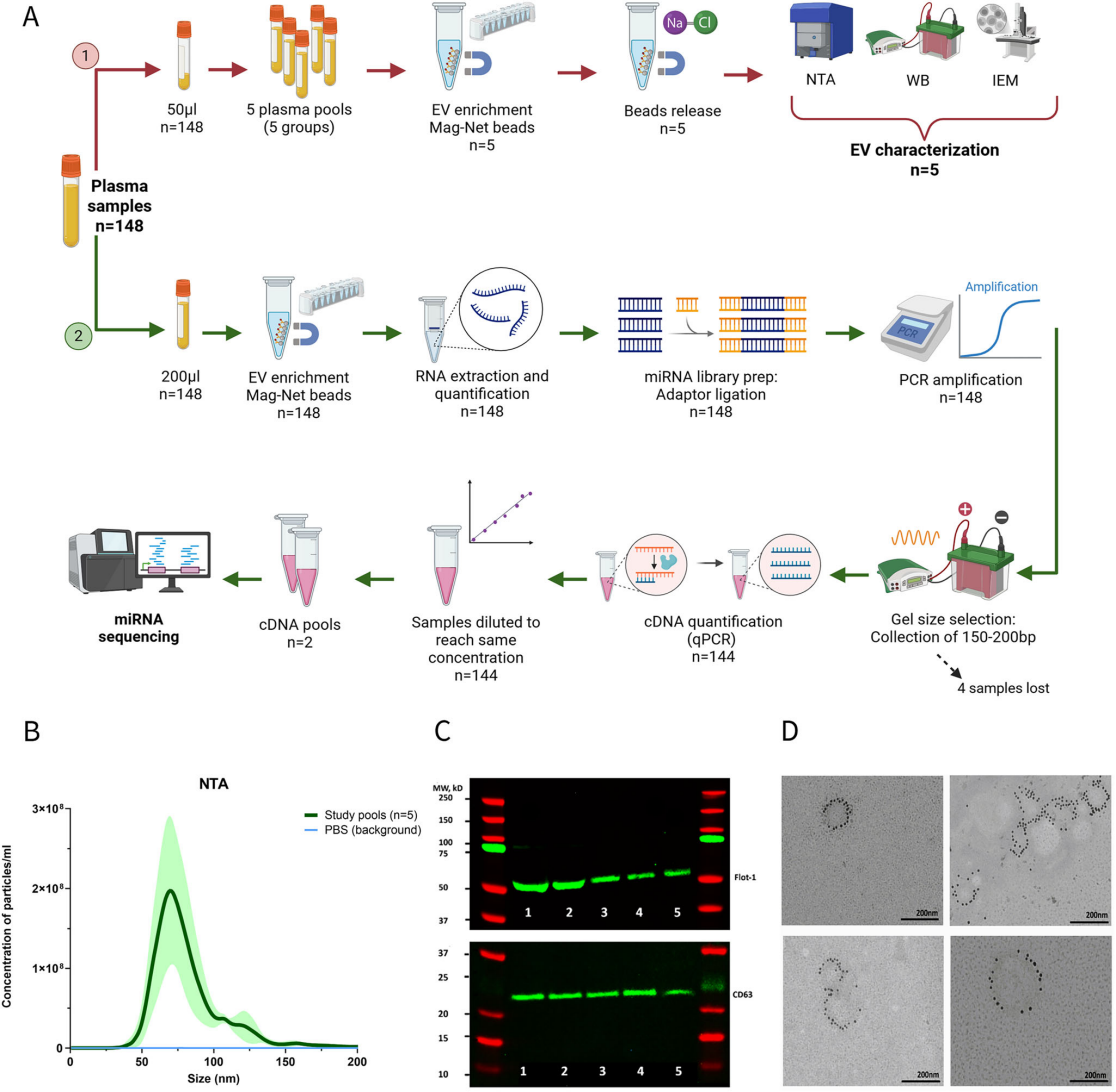
Method overview and EV characterization. (A) Method overview of 148 plasma samples (1), (red arrows) pooled in the five study groups for EV characterization, and (2), (green arrows) individually analysed for miRNA sequencing. Created in BioRender.com. EV characterization using (B) Nanoparticle tracking analysis (NTA) of EV‐enriched plasma pools (Study pools) and PBS control (PBS background) presented as mean ± SD (*n* = 5), (C) Western blot analysis (WB) showing the five plasma pools in each well: (1) Lean control group, (2) Obesity control group, (3) MASLD group at baseline, (4) 1 month, and (5) 5 months targeting Flot‐1 (MW ∼49 kDa), CD63 (MW ∼25‐60 kDa). (D) Representative immuno electron microscopy (IEM) image of CD9‐positive gold particles, scale bar 200 nm.

Originally, a total of 148 plasma samples were distributed as follows: Lean control group (*n* = 27), Obesity control group (*n* = 25), MASLD baseline (*n* = 36), MASLD 1 month (*n* = 30) and MASLD 5 months (*n* = 30) (Figure 2A). However, four samples (from different individuals) were lost during the gel size selection step in the miRNA library preparation, which resulted in changes in the final sample size (see Figure 1 for a detailed flowchart of the participant distribution). Consequently, all analyses presented in this study are based on this final sample distribution: Lean control group (*n* = 26), Obesity control group (*n* = 24), MASLD baseline group (*n* = 35). Longitudinal paired analysis of the MASLD intervention group included 28 individuals at baseline, 1 month and 5 months (Figure 1).

Data are presented according to the MASLD grouping, whereas data from the original design (NAFLD + MetS) are presented in the Supporting Information (Tables S4 and S5).

### 2.4 Quality Management System

All experimental procedures associated with EV isolation and miRNA analysis were conducted within an ISO17025 accredited (National Association of Testing Authorities, Australia) research facility. All data were recorded within a 21 Code of Federal Regulation Part 11 compliant electronic laboratory notebook (Lab Archives, Carlsbad, CA, USA).

### 2.5 EV Enrichment

For each plasma sample, 200 µL was used for EV enrichment and miRNA analysis, and an additional 50 µL was pooled within each group to create five plasma pools for EV validation (Figure 2A). EV enrichment was performed using the Mag‐Net isolation kit (MagReSynSAX, RESYN BIOSCIENCES, cat: #MR‐SAX005) following the manufacturer instructions and as previously described (Wu et al. 2024). In brief, 50 µL of MagReSyn beads was washed twice using 200 µL BTP equilibration buffer (50 mM Bis trip Propane pH 6.3, 150 mM NaCl) in a 96‐well plate (Deepwell plate, Eppendorf) and mixed gently for 30 s at 800 rpm on a ThermoMixer. The plate was placed on a magnetic stand (Invitrogen, 96‐well, lot: 2598090) for 5 min whereafter the equilibration solution was removed. Next, 200 µL of each plasma sample were diluted in 200 µL binding buffer in a 96‐well plate (0.2 non‐skirted Thermo‐Scientific) and mixed for 30 s at 800 rpm. The diluted plasma samples were transferred to the equilibrated beads´ plate and mixed for 30 min at 800 rpm at room temperature (RT). After incubation, the beads were washed three times with 500 µL BTP equilibration buffer and finally resuspended in 100 µL DPBS (Gibco, 500 mL, lot: 2931450), transferred to Eppendorf tubes and stored at −80°C for later RNA extraction.

For EV validation, EVs bound to the beads were released after the last wash by resuspension in 100 µL NaCl (25 mM Bis tris Propane pH 6.5, 1M NaCl) and placed on the mixer for 5 min at 800 rpm. Lastly, the plate was placed on the magnetic stand for 5 min, whereafter the supernatant was collected and transferred to Eppendorf tubes and stored at −80°C. These samples were used for EV validation by nanoparticles tracking analysis (NTA), Western blotting and Immuno Electron Microscopy (IEM).

### 2.6 Characterization of EVs

#### 2.6.1 Nanoparticle Tracking Analysis

The particle concentration and size distribution were measured using NTA (NanoSight NS500) in five isolated EV pools. Five videos were measured with a frame count of 749 using a light‐scattering Blue405 laser and analysed using the Nanosight NTA v3 software. The instrument was calibrated using Nanosphere size standards (Polystyrene latex microspheres 100 nm, cat no: 3100A, lot nr: 268426, Nom Diam 15 mL, mean diam 101nm±3 nm). The sCMOS camera was set to level 13, viscosity at 0.877963, the temperature at 25°C, slider shutter at 1232, slider gain at 175, shutter/ms at 30.8, duration of 30 s, with five repeats/well, one wash/well, and a syringe pump flow of 50. A PBS control was measured to see the background and the five samples were diluted in PBS to reach the optimal concentration for analysis with 20–100 particles/frame.

#### 2.6.2 Western Blot Analysis

The protein concentration of the five isolated EV pools and a plasma control sample was measured using a Pierce BCA Protein Assay kit. (ref:23225) (Fig. S1). Next, 30 µg protein for each of the five isolated EV pools and 0.15 µg protein for the plasma control (200 times diluted compared to the EV pools) was added to a Bolt 4%–12% Bis‐Tris Plus WedgeWell Gell (Invitrogen, ref: NW04120BOX) and run with 200 V for 25 min. The gel was transferred to a Wet Immobilon‐FL PVDF Membrane (Millipore, Ref: IPFL00010) using a Mini‐PROTEAN Tetra system at 100 V for 60 min. Hereafter, the membrane was washed using TBS‐TWEEN and incubated in the dark on a shaker for 60 min in 15 mL TBS Blocking Buffer (Intercept, Albumin, #927‐60001). The membrane was incubated overnight at 4°C with the primary antibodies: CD63 rabbit (Cell signalling, #52090, Molecular Weight (MW)∼25–60 kDa, diluted 1:500), and Flotillin rabbit (Cell signalling, #18634T, MW∼49 kDa, 1:1000) (separately), for checking EV enrichment and lipoprotein co‐isolation was checked using Anti‐Apolipoprotein A 1 Rabbit (Apo‐A1) (Abcam, #AB52945, MW∼28 kDa, DF1:1000). Next, the membranes were washed with washing buffer (20X TBS in diH20, with 1% TWEEN‐20) and incubated with the Goat Anti‐Rabbit secondary antibody (diluted 1:10000) (LI‐COR, #925‐32211). Lastly, the membranes were washed three times and measured using a ChemiDoc MP Imaging system (BIO‐RAD). (For more details, see the Supporting Information).

#### 2.6.3 Immuno Electron Microscopy

IEM was performed as previously described (Mellergaard et al. 2026; Ellegaard Nielsen et al. 2020). In brief, 5 µL of isolated EV pool was mounted onto carbon coated, glow discharged, 400 mesh Ni grids for 30 s (SPI Supplies, Chester, PA, USA) followed by three wash steps with two drops of PBS and blocking using three drops of 0.5% ovalbumin (pH 7.0, Sigma–Aldrich, St. Louis, MO, USA) in PBS. Next, the grids were incubated with the primary monoclonal mouse anti‐CD9 antibody (BD Pharmingen, San Diego, CA, USA, Cat. M‐L13) diluted 1:50 in 0.5% ovalbumin in PBS for 30 min at 37°C. Hereafter, three new PBS wash steps were performed followed by incubation with the secondary antibody 10 nm gold‐conjugated goat anti‐mouse (British BioCell, Cardiff, UK, cat: GMHL10, batch 23070115) diluted 1:25 in 0.5% ovalbumin in PBS for 30 min at 37°C. Next, the grids were washed again with three drops of PBS and blocked by incubating for 10 min at RT with three drops of 1% cold fish gelatine (Sigma–Aldrich, St. Louis, MO, USA). Last wash step with PBS was performed followed by staining with one drop of 0.5% phosphotungstic acid (Ted Pella, Caspilor AB, Lindingö, Sweden), (pH 7.0) for 30 s and blotted dry on filter paper. Finally, electron microscopy was performed using a JEM‐1400Flash electron microscope operated at 60 keV (JEOL, Tokyo, Japan) and the images were captured with a TVIPS TemCam FX416 digital camera (TVIPS, Gauting, Germany).

### 2.7 Next Generation Sequencing

RNA extraction was performed using the Norgen Biotek Exosomal RNA extraction kit for plasma (cat: 58000) following the manufacturer´s instructions. First, 100 µL of EV‐enriched sample was transported to a DNA LoBind tube and diluted in PBS to reach a total volume of 200 µL. RNA was quantified using a Qubit microRNA assay kit (Invitrogen, lot: 2507896) and measured using the Qubit 4 Fluorometer instrument (Invitrogen, Thermo Fisher Scientific, software version: APP 2.01 + MCU v0.26).

miRNA library preparation was performed using NEXTFLEX, small RNA‐Seq Kit v4 with Unique Dual Indices (UDIs) (PerkinElmer, V4, Cat # NOVA‐5132‐42 and #NOVA‐5132‐43), following the manufacturer's instructions. For PCR amplification, 4 µL of UDI barcoded primer mix and 6 µL of small RNA PCR master mix were added to the plate and incubated for 30 s at 98°C followed by 23 cycles of 10 s at 98°C, 20 s at 65°C, 15 s at 72°C, and lastly 2 min at 72°C, whereafter the samples were stored at −20°C.

Gel size selection and cleanup were performed using NEXTFLEX, a small RNA‐Seq Kit v3 with UDIs. Each PCR product was stained with 5.2 µL 6X gel loading dye (purple, no SDS, B70255, New England Biolabs), whereafter all samples and a low molecular weight control ladder were loaded onto a 6% TBE‐PAGE gel on a Mini gel Tank at 200 V for 30 min. Next, the gel was stained and incubated for 10 min on a shaker with 5 µL SYBR Gold (Nucleic acid stain, lot: 2700997, Invitrogen) diluted in 50 mL TBE 1X buffer. The samples were visualized using a UV transilluminator and cut between 150–200 bp. The gel piece was crushed and soaked overnight in IDTE pH 8 with 70 rpm agitation at RT. Beads cleanup was performed following the manufacturer's instructions, and the final sequencing library were collected and transferred to a 96‐well PCR plate and stored at ‐20°C overnight.

cDNA quantification was performed using a KAPA Library Quant Kit (Illumina, Universal qPCR mix, lot: 515766, KAPA Biosystems). Dilutions of 1:10000 and 1:50000 for each sample were created along with six DNA standards which were all run in duplicates through a qRT‐PCR cycle of 5 min at 95°C (1 cycle), 30 s at 95°C and 45 s at 60°C (35 cycles) and analysed using the QuantStudio Design & Analysis Software v1.5.3. The concentration of cDNA in each sample was calculated using the Ct value and standard curves (Figure S3). Finally, all samples were diluted to the same concentration and pooled in two pools. The prepared library was sent for miRNA sequencing at St. Lucia Campus (University of Queensland, Brisbane, Australia). (For more details, see Supporting Information).

### 2.8 Statistical Analysis

Small RNA sequencing data were analysed using miRDeep2 software (version 2.0.1.2) and modelled using a negative binomial linear model, and statistical significance of differential expression was assessed with a Wald test using the DESeq2 package in R (version 4.4.3). Pre‐filtering only included miRNAs with a raw count of five in at least ¼ of the samples. To test statistical significance, hierarchical cluster analysis and volcano plot were created. miRNAs with *p* values < 0.05 and log2FoldChange > 0.29 (fold change [FC] ≥ 1.22) were considered significantly differentially expressed. DESeq2 data were visualized as Variance Stabilizing Transformation (VST)‐normalized miRNA expression and compared between the three baseline groups, and a paired analysis was performed for the MASLD intervention group including only those who completed the 1‐ and 5‐months intervention (*n* = 28) (Figure 1). Linear regression analysis using the Benjamini–Hochberg (BH) method to correct for the False Discovery Rate (FDR) was performed for selected miRNAs and clinical parameters. Disease predictive ability was investigated using logistic regression analysis with an AUROC curve. Additionally, the sensitivity, specificity, positive predictive value (PPV) and negative predictive value (NPV) of a combined miRNA panel were calculated. A manual search of the MiRbase.org and GeneCaRNA.com databases identified related diseases. miRNA target genes were identified through validated databases miRTarBase, miRecords and TarBase using the multiMiR() function in R. Functional analysis with Gene Ontology (GO) and Kyoto Encyclopaedia of Genes and Genomes (KEGG) was performed using the clusterProfiler package in R. GO enrichment for biological processes was created using the *enrichGO()* function, and KEGG pathways using the *enrichKEGG*(), both with BH correction for multiple testing. Two new groups were created by first combining all the individuals from the MASLD baseline group (*n* = 35) with the obesity control group (*n* = 24) to create a new group called All individuals with obesity (*n* = 59), and then regrouping them according to the liver fat content as the 0.3 quantile (top 30% liver fat: 9.5%–33.1%, high liver fat group, *n* = 18), and the 0.7 quantile (bottom 30% liver fat: 1.6%–2.9%, low liver fat group, *n* = 18). For identification of markers for high liver fat, differential expression analysis was performed on the two groups (high vs. low), whereas the following logistic regression analysis was performed to discriminate the high liver fat group (*n* = 18) among all individuals with obesity (*n* = 59). Statistical significance was defined as *p *< 0.05 for all statistical analyses.

#### 2.8.1 Biomarker Analysis

To build a robust biomarker framework, we applied initial logistic regression (as described above), leave‐one‐out cross‐validation (LOOCV), and synthetic data augmentation using generative artificial intelligence (GAI), aiming to minimise overfitting and identify the most informative miRNA combinations. PyCaret's classification module was used to train 14 different algorithms for the two outcomes as previously described (Palma et al. 2025). In addition, the optimal sensitivity, specificity, PPV, NPV, AUROC curve, area under precision recall graph and Brier score were calculated for the training and test data. Finally, feature importance was calculated for the best model, providing an overall assessment of which biomarkers were most crucial in distinguishing between control and case samples.

## 3 Results

### 3.1 Characterization of Enriched EVs

NTA analysis of the five isolated EV pools revealed a mean particle concentration of 7.43 × 10^9^ particles/mL and a mean particle size of 83.96 nm (Figure 2B), suggesting enrichment of small EVs. A PBS control sample was included to visualize the background, which was 0.4 particles/frame and a concentration of 3.61 × 10^6^ particles/mL. Western blot analysis showed a positive signal for the EV markers Flotillin‐1 (Flot‐1), and CD63 (Figure 2C), and IEM confirmed the presence of CD9‐positive EVs (Figure 2D). Western blot analysis using APO‐A1 antibody showed a markedly decrease of lipoprotein contamination (Figure S2).

### 3.2 Distinct miRNA Expression Patterns in MASLD Compared With the Obesity Control Group

**FIGURE 3:**
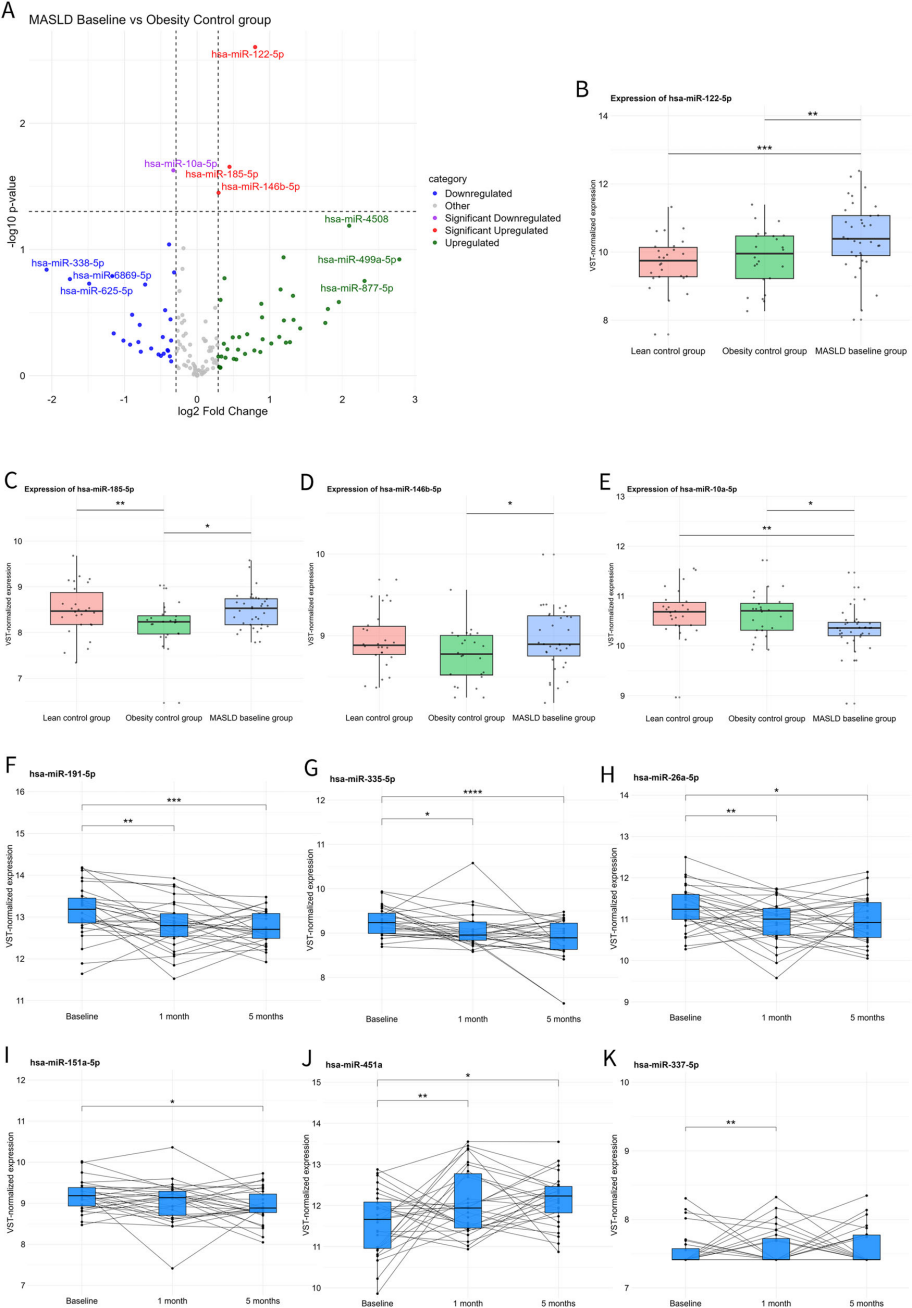
Volcano plot and group comparison of miRNA expression levels. (A) Volcano plot analysis illustrating differentially expressed miRNAs between MASLD and the Obesity control group at baseline, miRNAs with a *p* value < 0.05 and log2foldchange ≥0.29 (equivalent to FC ≥ 1.22) are considered significantly differentially expressed. miRNA VST‐normalized expression between the baseline groups is illustrated for (B) miR‐122, (C) miR‐185, (D) miR‐146b, (E) miR‐10a, whereas the effect of weight loss intervention for the MASLD group is illustrated with downregulated miRNAs (F) miR‐191, (G) miR‐335, (H) miR‐26a, and (I) miR‐151a, and upregulated miRNAs (J) miR‐451a, and (K) miR‐337 (*n* = 28) at Baseline, 1 month, and 5 months. A box plot illustrating each group and the individual participants as dots (Connected with a line for the intervention group). Data presented as median, interquartile range, whiskers and significant level: *p* < 0.05 (*), *p* < 0.01 (**), *p* < 0.001 (***), *p* < 0.0001 (****).

We found that EV‐based miR‐122 (*p* < 0.01), miR‐185 (*p < *0.05), and miR‐146b (*p < *0.05) were significantly upregulated, while miR‐10a (*p < *0.05) was significantly downregulated at baseline in the MASLD group compared to the obesity control group (Table 1 and Figure 3A–E) (combined as Panel 1). Furthermore, seven miRNAs were differentially expressed in the MASLD group compared to the lean control group, and seven in the obesity control group compared to lean control group (Table 1). Additionally, comparing levels between the MASLD group and the lean control group at baseline, miR‐122 was significantly upregulated (*p < *0.001, Figure 3B) and miR‐10a was significantly downregulated (*p < *0.01, Figure 3E). Surprisingly, miR‐185 was significantly upregulated in the lean control group compared to the obesity control group (*p < *0.01, Figure 3C). Finally, hierarchical cluster analysis revealed no clear clustering (Figure S4A, B).

**TABLE 1:** Differential expression analysis for the three groups at baseline.

MASLD versus obesity control group				
miRNA	*p* value	Log2FoldChange	Fold change	Regulated
hsa‐miR‐122‐5p	0.0025	0.7989	1.739	Up
hsa‐miR‐10a‐5p	0.0236	−0.3273	0.797	Down
hsa‐miR‐146b‐5p	0.0356	0.2951	1.227	Up
hsa‐miR‐185‐5p	0.0222	0.4458	1.362	Up

MASLD versus lean control group				
miRNA	*p* value	Log2FoldChange	Fold change	Regulated
hsa‐miR‐122‐5p	0.0001	1.0285	2.039	Up
hsa‐miR‐10a‐5p	0.0065	−0.3848	0.7658	Down
hsa‐miR‐10b‐5p	0.0291	−0.2926	0.8164	Down
hsa‐miR‐9985	0.0018	−1.3013	0.4057	Down
hsa‐let‐7i‐5p	0.0439	−0.4633	0.7253	Down
hsa‐miR‐378i	0.0449	3.1480	8.8642	Up
hsa‐miR‐331‐5p	0.0429	3.4761	11.1278	Up

Obesity control group versus lean control group				
miRNA	*p* value	Log2FoldChange	Fold change	Regulated
hsa‐miR‐16‐5p	0.0228	−0.3170	0.8027	Down
hsa‐miR‐486‐5p	0.0237	−0.4182	0.7483	Down
hsa‐miR‐185‐5p	0.0063	−0.5667	0.6751	Down
hsa‐let‐7i‐5p	0.0045	−0.7126	0.6102	Down
hsa‐miR‐205‐5p	0.0154	−4.5325	0.0432	Down
hsa‐miR‐378i	0.0117	4.2966	19.6519	Up
hsa‐miR‐378c	0.0437	3.5157	11.4375	Up

*Note*: Differential expression analysis of (1) MASLD versus obesity control group, (2) MASLD versus lean control group, and (3) obesity control group versus lean control group. miRNAs with a *p* value < 0.05 and log2foldchange ≥ 0.29 (equivalent to FC ≥ 1.22) are considered significantly differentially expressed.

### 3.3 Differentially Expressed miRNAs Decrease During Weight Loss Intervention in the MASLD Group

In the MASLD group during intervention, a comparison between 5 months and baseline revealed 14 differentially expressed miRNAs, 1 month versus baseline revealed 13, and lastly 5‐months versus 1 month revealed six differentially expressed miRNAs (Table 2 and Figure S5). Next, paired analysis of the miRNA expression levels during the intervention revealed a significant decrease in miR‐191 (*p < *0.01), miR‐335 (*p < *0.05) and miR‐26a (*p < *0.01) from baseline to 1 month (Figure 3F–H), while miR‐451a (*p < *0.01) and miR‐337 (*p < *0.01) were significantly upregulated (Figure 3J, K). Furthermore, expression levels of miR‐191 (*p < *0.001, Figure 3F), miR‐335 (*p < *0.0001, Figure 3G), miR‐26a (*p < *0.05, Figure 3H) and miR‐151a (*p* < 0.05 Figure 3I) decreased significantly from baseline to 5 months, while miR‐451a (*p < *0.05) was significantly upregulated (Figure 3J). A significant decrease in liver fat content and BMI in between all timepoints during weight loss intervention was observed (Figure S6A, B).

**TABLE 2:** Differential expression analysis for the MASLD group.

MASLD intervention: Downregulated miRNAs				MASLD intervention: Upregulated miRNAs			
MASLD 5 months versus MASLD baseline				MASLD 5 months versus MASLD baseline			
miRNA	*p* value	Log2 Fold change	Fold change	miRNA	*p* value	Log2 Fold change	Fold change
hsa‐miR‐335‐5p	0.0001	−0.681	0.624	hsa‐miR‐1260a	0.0023	2.926	7.598
hsa‐miR‐191‐5p	0.0004	−0.423	0.746	hsa‐miR‐374a‐5p	0.0070	3.004	8.024
hsa‐miR‐584‐5p	0.0010	−2.764	0.147	hsa‐miR‐125b‐5p	0.0101	1.102	2.147
hsa‐miR‐4433b‐5p	0.0091	−2.322	0.200	hsa‐miR‐451a	0.0110	0.509	1.423
hsa‐miR‐30d‐5p	0.0118	−0.436	0.739	hsa‐miR‐660‐5p	0.0138	3.022	8.123
hsa‐miR‐151a‐5p	0.0127	−0.503	0.705				
hsa‐miR‐26a‐5p	0.0134	−0.349	0.785				
hsa‐miR‐98‐5p	0.0235	−1.266	0.416				
hsa‐miR‐146a‐5p	0.0466	−0.250	0.841				

MASLD 1 month versus MASLD Baseline				MASLD 1 month versus MASLD Baseline			
hsa‐miR‐191‐5p	0.0016	−0.379	0.769	hsa‐miR‐337‐5p	0.0039	4.265	19.226
hsa‐miR‐26a‐5p	0.0033	−0.416	0.749	hsa‐miR‐451a	0.0085	0.527	1.441
hsa‐miR‐584‐5p	0.0048	−2.373	0.193	hsa‐miR‐99a‐5p	0.0233	0.327	1.255
hsa‐miR‐335‐5p	0.0146	−0.429	0.743	hsa‐miR‐100‐5p	0.0241	0.389	1.309
hsa‐miR‐93‐5p	0.0184	−1.597	0.330	hsa‐miR‐186‐5p	0.0392	0.979	1.971
hsa‐miR‐98‐5p	0.0192	−1.316	0.402				
hsa‐let‐7e‐5p	0.0200	−2.151	0.225				
hsa‐let‐7f‐5p	0.0299	−0.450	0.732				

MASLD 5 months versus MASLD 1 month				MASLD 5 months versus MASLD 1 month			
hsa‐miR‐30d‐5p	0.0002	−0.660	0.633	hsa‐miR‐31‐5p	0.0047	7.908	240.2
hsa‐miR‐4433b‐5p	0.0150	−2.179	0.221	hsa‐miR‐1260a	0.0053	2.687	6.442
				hsa‐miR‐324‐5p	0.0404	1.429	2.692
				hsa‐miR‐181a‐5p	0.0173	0.757	1.690

*Note*: Differential expression analysis in the MASLD intervention group: 5 months versus Baseline, 1 month versus Baseline, and 5 months versus 1 month. Table divided in down‐regulated (left) and up‐regulated (right) miRNAs. miRNAs with a *p* value < 0.05 and a log2foldchange > 0.29 (equivalent to FC ≥ 1.22) are considered significantly differentially expressed.

### 3.4 Identification of MASLD‐ and HCC‐Related miRNAs in the MASLD Group

**FIGURE 4:**
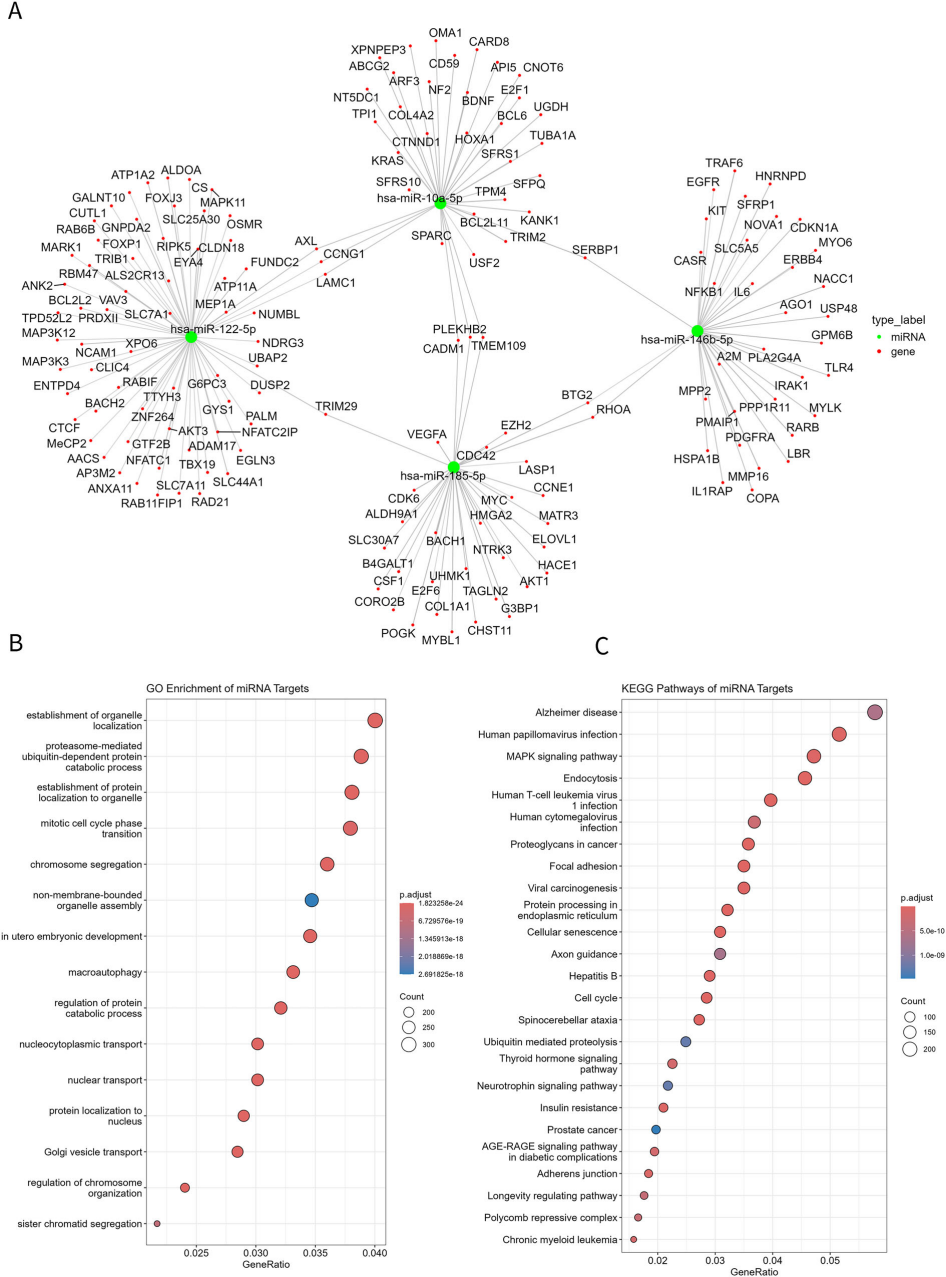
miRNA functional analysis. Functional analysis for the four significantly differential expressed miRNAs between the MASLD group and obesity control group: miR‐122, miR‐146b, miR‐10a, and miR‐185 are visualized as (A) miRNA target gene network using miRTarBase, miRecords, and TarBase, miRNA shown as nodes (green) and gene node (red) connected with regulatory interaction lines (grey). (B) GO and C) KEGG analysis visualized as dot plots with colour and size reflecting statistical significance and gene count, respectively.

A manual search of miRbase.org and GeneCaRNA.com revealed that miR‐122, miR‐26a, miR‐151a and miR‐30d were related to NAFLD or MASLD, and miR‐122, miR‐185, miR‐191, miR‐26a, miR‐146a, miR‐151a, miR‐584 and miR‐451 were associated with HCC (Table S1). Moreover, target gene network analysis for panel 1 revealed a high abundance of related target genes for each miRNA, with overlap between CCNG1, AXL, LAMC1, TRIM29, SERBP1, BTG2, RHOA, PLEKHB2, CADM1 and TMEM109 (Figure 4A). The biological functions of EV‐based miRNAs were investigated using GO and KEEG analyses, using the four significantly differentially expressed miRNAs in Panel 1. GO enrichment analysis showed that establishment of organelle and protein localization and proteasome‐mediated ubiquitin‐dependent protein catabolic process differed between the MASLD group and the obesity control group (Figure 4B). Finally, the KEGG enrichment revealed involvement of pathways regulating insulin resistance (IR), AGE‐RAGE signalling in diabetic complications, multiple neurological diseases and several cancer‐related pathways including the Mitogen‐Activated Protein Kinase (MAPK) pathway (Figure 4C).

### 3.5 MiR‐122 Correlates With Liver Fat Content and Liver Enzymes

**FIGURE 5:**
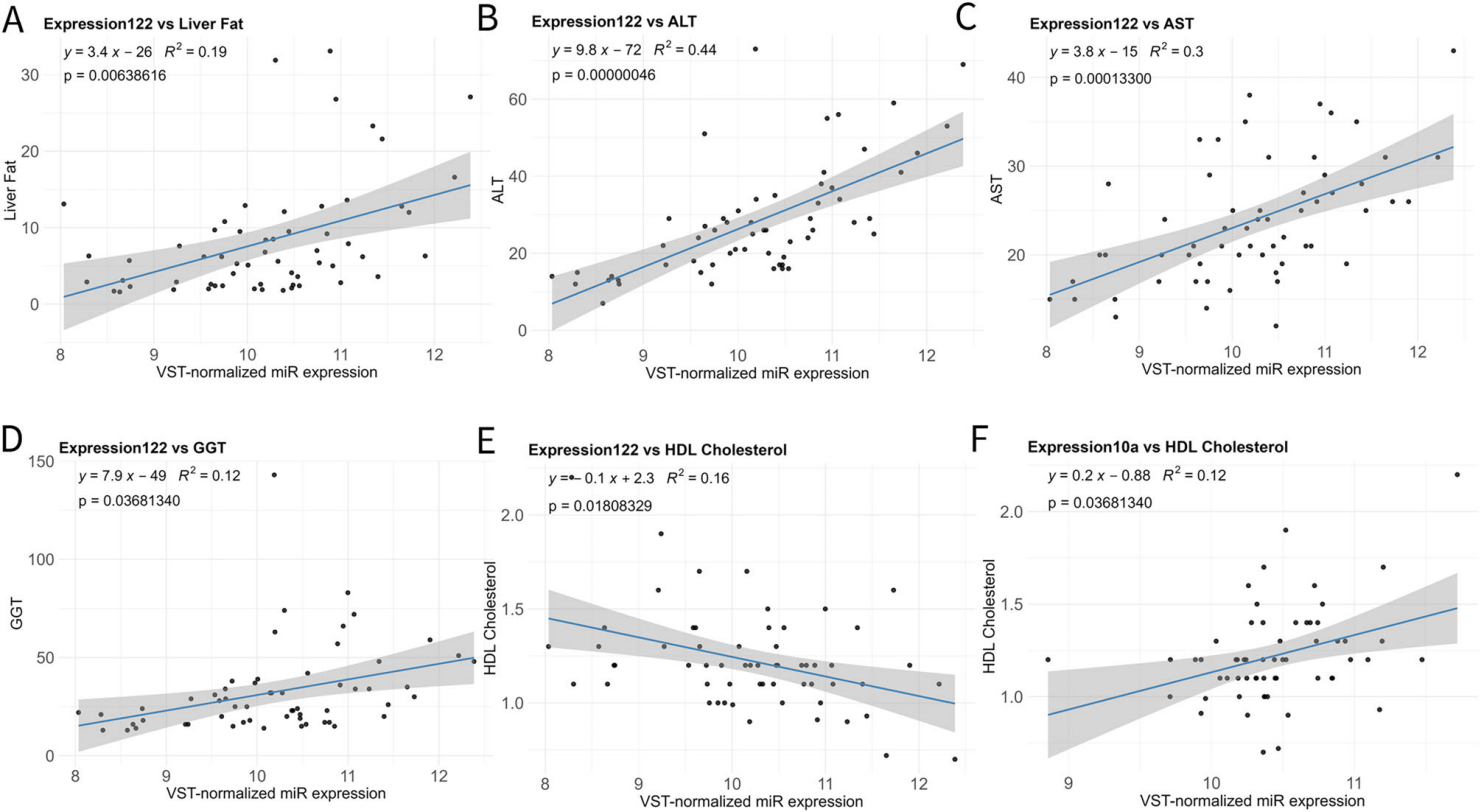
Linear regression between miRNA expression and clinical parameters. Linear regression analysis with BH correction showcasing VST‐normalized expression (X‐axis) of miR‐122 and clinical parameters (Y‐axis) for (A) liver fat (PDFF, %), (B) ALT levels units per litre (U/L), (C) AST levels (U/L), (D) GGT levels (U/L), (E) HDL cholesterol (mmol/L), and F) miR‐10a and HDL levels (mmol/L) among all individuals with obesity (*n* = 59). Data are presented as *R* ^2^ value and *p* value with significance level at *p* < 0.05.

Correlations between the individual miRNAs in panel 1 and the selected clinical parameters were assessed using linear regression analysis in all individuals with obesity (Table S2). The expression levels of miR‐122 correlated positively with liver fat content (*R^2^* = 0.19, *p < *0.01), ALT (*R^2^ = *0.44*, p < *0.000001), AST (*R* ^2^ = 0.3*, p < *0.001), GGT (*R^2^* = 0.12, *p < *0.05) and negatively correlated with HDL cholesterol (*R^2^ = *0.16*, p < *0.05, Figure 5A–E). Lastly, miR‐10a correlated positively with HDL cholesterol (*R^2^ = *0.12*, p < *0.05, Figure 5F). No significant correlations were observed for the remaining differentially expressed miRNAs and the selected clinical parameters (Table S2).

### 3.6 Combined miRNA Panels Showed Better Discrimination Compared to Individual miRNAs

#### 3.6.1 Prediction of MASLD Among all Individuals With Obesity Using Panel 1

**FIGURE 6:**
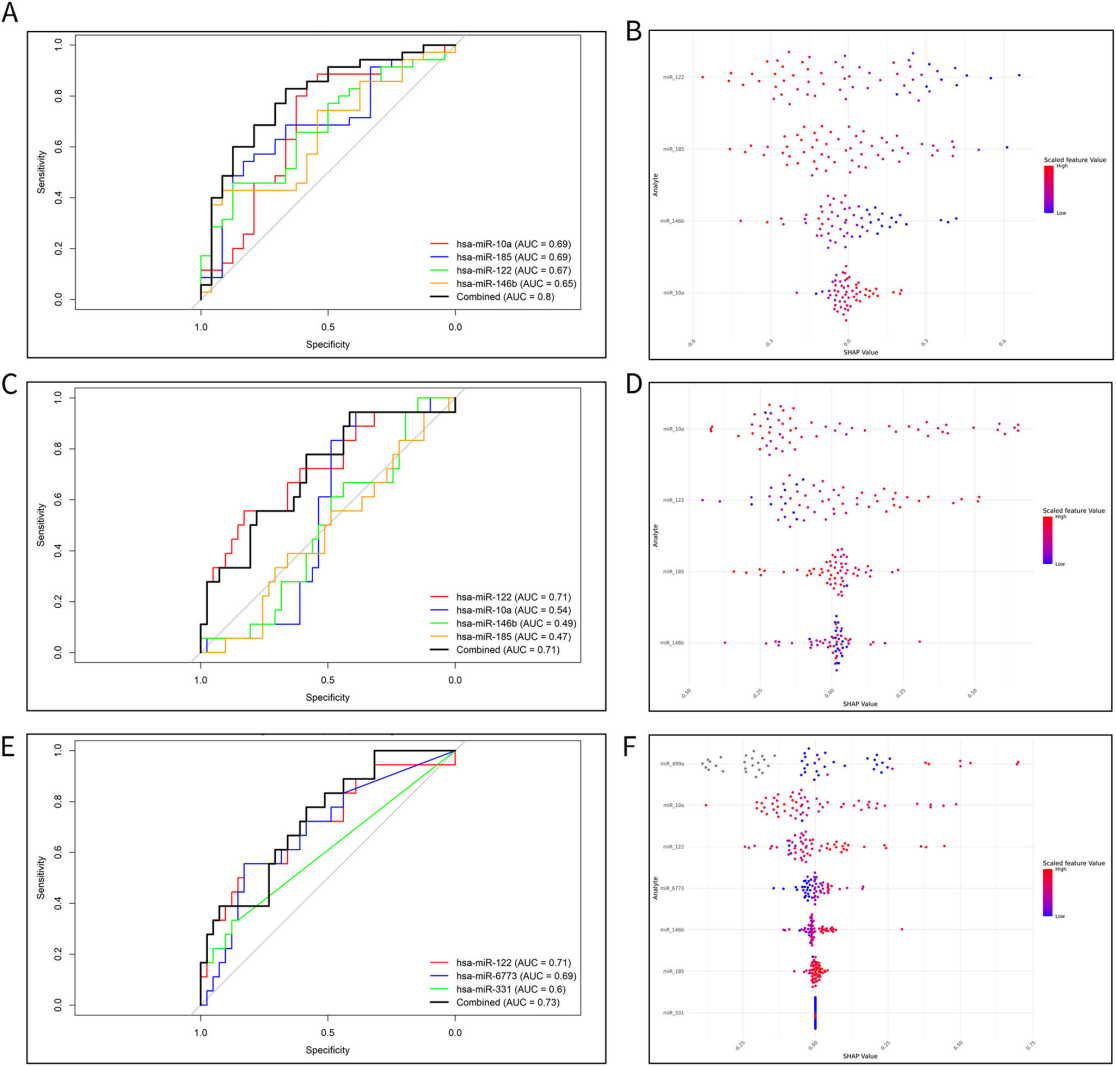
miRNA prediction of MASLD and high liver fat content. Logistic regression analysis with a ROC curve, and leave‐one‐out cross‐validation model with a SHAP analysis was created to (A+B) predict MASLD among all individuals with obesity (*n* = 59) using panel 1, (C+D) predict high liver fat (≥9.5%) among all individuals with obesity (*n* = 59) using panel 1, (E) using panel 3, and (F) using panel 2.

Initial logistic regression analysis showed that a combined miRNA panel 1 achieved higher discrimination (AUC = 0.80) of MASLD among individuals with obesity than single miRNAs (miR‐10a: 0.69; miR‐185: 0.69; miR‐122: 0.67; miR‐146b: 0.65) (Figure 6A). Combined panel 1 showed a sensitivity of 0.83, specificity 0.67, PPV 0.78 and NPV 0.73. When incorporating the estimated MASLD prevalence among individuals with obesity (75%) in the general population, the PPV increased to 0.88.

Using LOOCV classification, the combined miRNA panel 1 consistently outperformed single miRNAs across the receiver operating characteristics (ROC) (Table S3), precision–recall and calibration metrics (Fig. S7A), with the combined model achieving an AUROC of 0.75 and balanced sensitivity (75%) and specificity (74%). Furthermore, synthetic samples generated using GAI did not enhance model performance, as ROC curves from augmented datasets overlapped with those from the original data (Figure S7D). SHAP analysis identified miR‐122 and miR‐185 as the strongest contributors to the MASLD classification (Figure 6B).

#### 3.6.2 MiR‐122 Revealed the Highest Predictive Ability for High Liver Fat Content in Individuals With Obesity

Next, we assessed whether EV‐based miRNAs could identify individuals with a high liver fat content among all individuals with obesity (*n* = 59). Logistic regression using Panel 1 yielded acceptable discrimination (AUC = 0.71), driven predominantly by miR‐122 (AUC = 0.71), while miR‐10a (0.54), miR‐146b (0.49) and miR‐185 (0.47) contributed minimally (Figure 6C).

Using LOOCV, the combined panel again performed better than individual miRNAs across the ROC (Table S3), precision–recall and calibration curves (Figure S7B), with the combined panel providing an acceptable discrimination (AUROC 0.72), with relatively high sensitivity (78%) but lower specificity (59%). GAI did not improve predictive performance (Figure S7E). SHAP analysis indicated that miR‐122 were the main driver of classification (Figure 6D).

#### 3.6.3 Comparison of miRNA Panels for Discrimination of High Liver Fat

Differential expression analysis comparing high liver fat (top 30%, *n* = 18) and low liver fat (lower 30%, *n* = 18) sub‐groups identified seven significantly differentially expressed miRNAs (Panel 2): six upregulated (miR‐122, miR‐185, miR‐6773, miR‐499a, miR‐146b, miR‐331) and one downregulated (miR‐10a) (Table 3 and Figure S7F). Based on significance and biological plausibility, three miRNAs (miR‐122, miR‐6773 and miR‐331) were selected to construct a refined biomarker Panel 3. Again, we assessed whether EV‐based miRNAs could identify individuals with high liver fat content among all individuals with obesity (*n* = 59). Panel 3 demonstrated improved discrimination for high liver fat (AUC = 0.73) compared to individual miRNAs (Figure 6E).

**TABLE 3:** Differential expression analysis for high versus low liver fat groups.

High liver fat versus low liver fat				
miRNA	*p* value	Log2 Fold change	Fold change	Regulated
hsa‐miR‐122‐5p	0.000051	1.432	2.698	Up
hsa‐miR‐185‐5p	0.017056	0.623	1.540	Up
hsa‐miR‐6773‐5p	0.025813	1.865	3.643	Up
hsa‐miR‐499a‐5p	0.033633	3.568	11.861	Up
hsa‐miR‐146b‐5p	0.038328	0.336	1.262	Up
hsa‐miR‐331‐5p	0.046776	3.642	12.484	Up
hsa‐miR‐10a‐5p	0.032697	−0.348	0.786	Down

*Note*: High versus low liver fat. Differential expression analysis between high liver fat group (*n* = 18) and low liver fat group (*n* = 18). miRNAs with a *p* value < 0.05 and log2foldchange ≥ 0.29 (equivalent to FC ≥ 1.22) are considered significantly differentially expressed.

LOOCV ROC and precision–recall analyses showed enhanced performance of the combined panel 2 with achieving an AUROC of 0.89, perfect sensitivity (100%) and moderate specificity (63%), along with the highest AUPRC (0.81) and a favourable Brier score (0.21) (Table S3). Furthermore, the calibration curves showed appropriate alignment between predicted and observed probabilities (Fig. S7C). As before, the GAI did not improve model robustness (Figure S7G). SHAP analysis showed that miR‐122 was the dominant predictor, followed by miR‐6773 and miR‐331 (Figure 6F).

## 4 Discussion

The clinical management of MASLD remains challenging, particularly in individuals with obesity, where early identification of those at the highest risk for progressive liver disease is hindered by the absence of applicable, non‐invasive biomarkers. Current diagnostic tools, including liver enzymes, imaging and liver biopsy, either lack sensitivity, are costly, or are unsuitable for population‐level screening. To address this need, we evaluated the diagnostic performance of EV‐derived miRNAs as candidate biomarkers to identify MASLD or higher liver fat content in individuals with obesity. Despite the complexity of their biology, several miRNAs have been identified as key operators in liver pathogenesis (Carpi et al. 2024). The main focus of this field has been the discovery of circulating miRNAs from which several studies have identified circulating miR‐122 as a potential biomarker for NAFLD and MASLD (Tobaruela‐Resola et al. 2025; Yamada et al. 2013; Tobaruela‐Resola et al. 2024).

### 4.1 EV‐Based miRNAs as Potential Biomarkers for MASLD

We found that the liver‐related miR‐122 was significantly upregulated in the MASLD group at baseline compared to the obesity control group. In line with our findings, only a single previous study has been published on the identification of EV‐based miRNA profiles, including miR‐122 in MASLD patient samples (Caviglia et al. 2025).

Similar to our study, they highlighted the shift toward an EV‐based approach due to their higher stability and cell specificity, thus potentially making them more suitable biomarker candidates. miR‐122 represent the most abundant liver‐related miRNA accounting for approximately 70% of total liver miRNAs (Hochreuter et al. 2022; Becker et al. 2015). Circulating miR‐122 levels increase early during MASLD development and decrease during disease progression (Hochreuter et al. 2022; Erceg et al. 2025). miR‐122 is released in response to hepatocyte damage and is suggested to be involved in hepatic cholesterol and lipid metabolism, thus playing a fundamental role in liver homeostasis maintenance (Szabo and Bala 2013). Gene network analysis in the present study revealed numerous target genes for miR‐122, including CNNG1, ADAM17, ALDOA, SLC7A1 and CUTL1 which are involved in hepatocarcinogenesis, epithelial mesenchymal transition and cell proliferation (Colaianni et al. 2024; Nakao et al. 2014). Furthermore, the lipogenic enzyme Sirturin‐1 (SIRT1) is a key miR‐122 targets in lipid metabolism (Long et al. 2019) and has been proposed to play a protective role in NAFLD (Long et al. 2019; Nassir and Ibdah 2016). miR‐122 repress the LKB1/AMPK pathway through downregulation of SIRT1, which leads to the upregulation of lipid droplets in hepatocytes and induces steatosis and lipogenesis in NAFLD (Long et al. 2019). We found that mir‐122 correlated significantly with liver fat content, liver enzymes (ALT, AST, GGT) and HDL cholesterol (some of which are related to the definition of MASLD (Chan et al. 2023)). These results are in line with previous literature showing positive correlations between circulating miR‐122 and ALT levels in NAFLD patients (Pirola and Gianotti 2015) and in HCC patients (Ruoquan et al. 2014). Collectively, these data suggest that miR‐122 is associated with ongoing liver damage/pathogenesis and could be a potential biomarker for MASLD development, although further research is needed to substantiate this.

Furthermore, studies have shown a significant upregulation of miR‐146b in the whole blood of patients with NAFLD (Aghajanzadeh et al. 2023) as well as in rodent obesity/NAFLD models (Zhang et al. 2020; Chartoumpekis et al. 2012). Our gene network analysis demonstrated a link between miR‐146b and TRAF6/IRAK1, a well‐known axis in the NF‐KB pathway (Lou et al. 2023), which plays a distinct role in liver disorders, and inhibition of this pathway prevents HSC activation and decrease inflammation (Aghajanzadeh et al. 2023). Additionally, miR‐185 is related to NAFLD through enhanced lipid metabolism (Tan et al. 2022) and affects IR in mouse models (Wang et al. 2014). Interestingly, a study showed interaction between miR‐185 and the CDC42 gene, which is a key regulator of the MAPK pathway (Ma et al. 2022). Finally, we found a significant downregulation of miR‐10a in the MASLD group. Although few studies have previously investigated miR‐10a in the context of MASLD, one study reported significant negative correlation between liver fat and miR‐10a expression (Quintás et al. 2022). Moreover, our gene network analysis revealed multiple regulatory interactions among miR‐122, miR‐185, miR‐146b and miR‐10a. Several of these genes have been investigated in HCC‐focused studies, where CCNG1 and BTG2 are associated with the tumour suppressor gene p53 (Fornari et al. 2009; Zhang et al. 2011) and LAMC1, CADM1 and Axl, which are involved in the Akt pathway (Ye et al. 2019; Wang et al. 2019; Breitenecker et al. 2024). Thus, the miRNAs identified in this study has previously been associated with liver fat and cancer‐related pathways, which highlights their biomarker potential and possible involvement in the pathogenesis of MASLD. However, more research is needed on the specificity of these miRNAs in MASLD development and progression to more clearly describe their functions and impact.

### 4.2 Clinical Perspective

This study revealed a good biomarker potential of the combined miRNA panel 1 with high sensitivity and better discrimination of MASLD among all individuals with obesity compared with the individual miRNAs. Additionally, when the prevalence of 75% for MASLD among individuals with obesity in the general population was incorporated in the analysis, this revealed an increase in PPV, thus further highlighting its potential clinical value. Moreover, as recently reviewed, MASH has a prevalence of 34% among individuals with obesity and HCC has an incidence rate of 14.45 per 1000 person‐years among those with MASLD and advanced fibrosis (Younossi et al. 2024). Thus, identifying individuals with high liver fat content is important because of the likely increased risk of progression. This study highlights the combined miRNA panel 2 and 3, especially miR‐122, which revealed a good predictive ability for high liver fat content among all individuals with obesity. To the best of our knowledge, no research involving miR‐6773 has been conducted, whereas, miR‐331 has been documented as a promoter of the proliferation, migration and invasion of tumour cells in HCC (Chang et al. 2014). This study shows that miR‐122 and miRNA combinations are promising predictors of MASLD and high liver fat content offering minimally invasive biomarkers for better management and early diagnosis.

### 4.3 Weight Loss Intervention

Younossi et al. predicted that without intervention, the prevalence of MASLD would increase with 6%–20%, MASH by 20%–35% and HCC by 65%–100% by 2030 (Younossi et al. 2024). Weight loss intervention has been suggested as the first‐line treatment to prevent such increases (Li et al. 2024). A strength of the present study is the personalized weight loss intervention, which resulted in a significant decrease in liver fat content, BMI and a decrease in several metabolic parameters such as ALT, AST and LDL as previously described (Askeland A, unpublished data, February 2026). To the best of our knowledge our study is the first to investigate EV‐based miRNAs in MASLD before and during weight loss intervention with assessments already after 1 month, revealing potential early responses to weight loss. Among the differentially expressed miRNAs observed in the MASLD group, several were related to MASLD, NAFLD, or HCC. A significant decrease during weight loss intervention was observed for miR‐191, miR‐26a, mir‐151a and miR‐335, all of which have been associated with the pathogenesis of several cancers, including HCC (Nagpal and Kulshreshtha 2014; Ali et al. 2018; Ebrahimi et al. 2023). Moreover, the results are in line with the current literature, showing the association of miR‐191 with HCC features such as hepatocyte balloon degeneration (Ezaz et al. 2020), and demonstrating miR‐335 ability to influence NAFLD and NASH through modulation of IR (Wu et al. 2024). Additionally, during the weight loss intervention in the present study, several miRNAs were significantly upregulated including miR‐451a, miR‐31, miR‐337, miR‐1260 and miR‐374a. This is supported by other studies, which revealed that miR‐451a, miR‐31 and miR‐337 are tumour suppressor miRNAs and have been shown to inhibit HCC progression (Cui et al. 2018; Du et al. 2017; Huang et al. 2015). Altogether, our findings support the involvement of specific miRNA profiles in the liver pathophysiology of MASLD.

Lastly, miR‐122 did not decrease significantly during the intervention, possibly because miR‐122 is known to increase during early MASLD and decrease again with disease progression (Hochreuter et al. 2022; Erceg et al. 2025). We speculate that although we observed significant improvements in several clinical parameters, including liver fat content, BMI and liver‐derived EV numbers as previously described (Mellergaard et al. 2026), these might precede changes in EV‐based miR‐122 expression. In future studies it would be particularly interesting to follow the expression pattern of miR‐122 from early MASLD to severe MASH or HCC to potentially identify the point of shift in expression, as well as performing miRNA profiling in liver‐derived EVs from patients with MASLD.

### 4.4 Limitations of the Study

This study was limited by the relatively small number of participants, and the change in study design from the original NAFLD+Mets to MASLD, which resulted in three individuals being moved from the obesity control group to the MASLD group thus, they did not receive personalized weight loss intervention and were therefore only represented in the baseline measurements. This case‐control study was the first to identify EV‐based miRNA profiles in patients with MASLD before and during personalized weight loss intervention; however, it was limited by the lack of a weight loss control group. Additionally, as this study represents a secondary analysis of the registered MULTISITE clinical trial (ClinicalTrials.gov NCT05699863), an independent validation cohort would require a separate clinical trial with a comparable design and population. According to the FDA‐NIH Biomarker Working Group (BEST) framework, clinical validation, including evaluation of diagnostic performance in independent cohorts, follows analytical validation of the assay and establishment of the intended use of the test. The present study constitutes an early‐phase biomarker discovery analysis; the LOOCV and synthetic data augmentation via GAI described in the Methods were specifically employed to minimise overfitting and maximise the generalisability of findings within the constraints of the available dataset. Future independent clinical validation studies will be required to confirm the diagnostic performance and clinical utility of the identified miRNA panels. It is also important to acknowledge that plasma lipoproteins circulate at concentrations approximately 10 million‐fold greater than EVs, meaning that complete separation of EVs from lipoproteins is technically unachievable with any current isolation method; all EV isolation protocols should therefore be considered enrichment procedures rather than absolute purification (Holcar et al. 2020; Johnsen et al. 2019; Simonsen 2017). The co‐isolation of EVs with lipoproteins is a well‐documented phenomenon, and their functional interactions in plasma have been extensively described. This inherent limitation of EV research must be considered when interpreting miRNA cargo data derived from EV‐enriched fractions.

## 5 Conclusion

In conclusion, this study identified that the liver‐ and MASLD‐related miR‐122, along with three other miRNAs, were differentially expressed in the MASLD group compared with the obesity control group at baseline. In addition, miR‐122 was significantly correlated with liver fat content and liver enzymes among all individuals with obesity. Furthermore, the combined miRNA panel 1 revealed good MASLD predictive ability, whereas panels 2 and 3 showed good discrimination of high liver fat content among all individuals with obesity. Lastly, this study identified a significant decrease in several MASLD and HCC related miRNAs during personalized weight loss intervention, thus altogether providing clear clinical potential as non‐invasive EV‐based biomarkers for MASLD diagnosis.

## Author Contributions

**Katja Lund Cliff**: Conceptualization, data curation, formal analysis, funding acquisition, investigation, methodology, project administration, validation, visualization, writing the original draft, writing – review and editing. **Domonic Guanzon**: Data curation, software, writing – review and editing. **Andrew Lai**: Data curation, software, writing – review and editing. **Katherin Scholz‐Romero**: writing – review and editing. **Gunna Christiansen**: investigation, methodology, writing – review & editing. **Aase Handberg**: conceptualization, funding acquisition, methodology, project administration, resources, supervision, writing – review and editing. **Carlos Salomon**: conceptualization, data curation, formal analysis, funding acquisition, methodology, project administration, resources, supervision, writing – review and editing. **Maiken Mellergaard**: conceptualization, funding acquisition, methodology, project administration, resources, supervision, writing – review and editing. All authors have read and agreed to the published version of the manuscript.

## Funding

The Independent Research Fund Denmark (10.46540/3101‐00394B), the Novo Nordisk Foundation (NNF17SA0031406, The Doctoral School in Medicine—Biomedical Science and Technology (Aalborg University), A.P. Møller og Hustru Chastine Mc‐Kinney Møllers Fond til almene Formaal (2024‐01094) and the Knud Højgaard Foundation (25‐02‐0265). *CS is supported by the National Health and Medical Research Council (NHMRC 1195451, Australia).

## Conflicts of Interest

The authors report no conflict of interest.

## Supporting information




**Supporting Information**: jex270173‐sup‐0001‐SuppMat.docx

**Supporting Information**: jex270173‐sup‐0002‐SuppMat.docx

## Data Availability

The raw data from this study is available upon request from Dominic Guanzon and the corresponding author Carlos Salomon: DOI: https://doi.org/10.48610/aaba5e5. However, restriction apply to the availability of the metadata to preserve patient confidentiality, thus is only available on request from the contact person of the clinical study, Aase Handberg.

## References

[jex270173-bib-0001] Aghajanzadeh, T. , M. Talkhabi , M. R. Zali , B. Hatami , and K. Baghaei . 2023. “Diagnostic Potential and Pathogenic Performance of Circulating miR‐146b, miR‐194, and miR‐214 in Liver Fibrosis.” Non‐Coding RNA Research 8, no. 4: 471–480. 10.1016/j.ncrna.2023.06.004. 37434946 PMC10331815

[jex270173-bib-0002] Ali, O. , H. A. Darwish , K. M. Eldeib , and S. A. Abdel Azim . 2018. “miR‐26a Potentially Contributes to the Regulation of Fatty Acid and Sterol Metabolism In Vitro Human HepG2 Cell Model of Nonalcoholic Fatty Liver Disease.” Oxidative Medicine and Cellular Longevity 2018: 8515343. 10.1155/2018/8515343. 30402207 PMC6196797

[jex270173-bib-0003] Askeland, A. , R. W. Rasmussen , M. Gjela , et al. 2025. “Non‐Invasive Liver Fibrosis Markers Are Increased in Obese Individuals With Non‐Alcoholic Fatty Liver Disease and the Metabolic Syndrome.” Scientific Reports 15, no. 1: 1–13. 10.1038/s41598-025-85508-y. 40148373 PMC11950363

[jex270173-bib-0004] Ban, E. , and E. J. Song . 2022. “Considerations and Suggestions for the Reliable Analysis of miRNA in Plasma Using qRT‐PCR.” Genes 13, no. 2: 328. 10.3390/genes13020328. 35205372 PMC8872398

[jex270173-bib-0005] Becker, P. P. , M. Rau , J. Schmitt , et al. 2015. “Performance of Serum microRNAs‐122,‐192 and‐21 as Biomarkers in Patients With Non‐Alcoholic Steatohepatitis.” PLOS ONE 10, no. 11: e0142661. 10.1371/journal.pone.0142661. 26565986 PMC4643880

[jex270173-bib-0006] Breitenecker, K. , D. Heiden , T. Demmer , et al. 2024. “Tumor‐Extrinsic Axl Expression Shapes an Inflammatory Microenvironment Independent of Tumor Cell Promoting Axl Signaling in Hepatocellular Carcinoma.” International Journal of Molecular Sciences 25, no. 8: 4202. 10.3390/ijms25084202. 38673795 PMC11050718

[jex270173-bib-0007] Byrne, C. D. , and G. M. Targher . 2024. “MASLD, MAFLD, or NAFLD Criteria: Have We Re‐Created the Confusion and Acrimony Surrounding Metabolic Syndrome?” Metabolism and Target Organ Damage 4, no. 2: 7. 10.20517/mtod.2024.06.

[jex270173-bib-0008] Carpi, S. , S. Daniele , F. Jacqueline , and D. Gabbia . 2024. “Recent Advances in miRNA-Based Therapy for MASLD/MASH and MASH-Associated HCC.” International journal of molecular sciences 25, no. 22: 12229. 10.3390/ijms252212229. 39596297 PMC11595301

[jex270173-bib-0009] Caviglia, G. P. , E. Casalone , C. Rosso , et al. 2025. “Extracellular Vesicles miRNome Profiling Reveals miRNAs Engagement in Dysfunctional Lipid Metabolism, Chronic Inflammation and Liver Damage in Subjects With Metabolic Dysfunction‐Associated Steatotic Liver Disease.” Alimentary Pharmacology & Therapeutics 62, no. 1: 22–32. 10.1111/apt.70150. 40208030 PMC12151544

[jex270173-bib-0010] Chan, W. K. , K. H. Chuah , R. B. Rajaram , L. L. Lim , J. Ratnasingam , and S. R. Vethakkan . 2023. “Metabolic Dysfunction‐Associated Steatotic Liver Disease (MASLD): A State‐of‐the‐Art Review.” Journal of Obesity & Metabolic Syndrome 32, no. 3: 197–213. 10.7570/jomes23052. 37700494 PMC10583766

[jex270173-bib-0011] Chang, R. M. , H. Yang , F. Fang , J. F. Xu , and L. Y. Yang . 2014. “MicroRNA‐331–3p Promotes Proliferation and Metastasis of Hepatocellular Carcinoma by Targeting PH Domain and Leucine‐Rich Repeat Protein Phosphatase.” Hepatology 60, no. 4: 1251–1263. 10.1002/hep.27221. 24825302

[jex270173-bib-0012] Chartoumpekis, D. V. , A. Zaravinos , P. G. Ziros , et al. 2012. “Differential Expression of microRNAs in Adipose Tissue After Long‐Term High‐Fat Diet‐Induced Obesity in Mice.” PLOS ONE 7, no. 4: e34872. 10.1371/journal.pone.0034872. 22496873 PMC3319598

[jex270173-bib-0013] Cheng, L. , R. A. Sharples , B. J. Scicluna , and A. F. Hill . 2014. “Exosomes Provide a Protective and Enriched Source of miRNA for Biomarker Profiling Compared to Intracellular and Cell‐Free Blood.” Journal of Extracellular Vesicles 3, no. 1: 1–14. 10.3402/jev.v3.23743. PMC3968297 24683445

[jex270173-bib-0014] Chooi, Y. C. , C. Ding , and F. Magkos . 2019. “The Epidemiology of Obesity.” Metabolism 92: 6–10. 10.1016/j.metabol.2018.09.005. 30253139

[jex270173-bib-0015] Colaianni, F. , V. Zelli , C. Compagnoni , et al. 2024. “Role of Circulating microRNAs in Liver Disease and HCC: Focus on miR‐122.” Genes 15, no. 10: 1313. 10.3390/genes15101313. 39457437 PMC11507253

[jex270173-bib-0016] Cui, H. , R. Song , J. Wu , W. Wang , X. Chen , and J. Yin . MicroRNA‐337 Regulates the PI3K/AKT and Wnt/β‐Catenin Signaling Pathways to Inhibit Hepatocellular Carcinoma Progression by Targeting High‐Mobility Group AT‐Hook 2. 2018;8, no. 3: 405–421. PMC5883092 29636997

[jex270173-bib-0017] Du, Z. , S. Niu , X. Xu , and Q. Xu . 2017. “MicroRNA31‐NDRG3 Regulation Axes Are Essential for Hepatocellular Carcinoma Survival and Drug Resistance.” Cancer Biomarkers 19, no. 2: 221–230. 10.3233/CBM-170568. 28269758 PMC13020714

[jex270173-bib-0018] Ebrahimi, A. , S. M. Derakhshan , D. Ghavi , Z. Foruzandeh , and S. Hashemi . 2023. “The Role of Mir‐151a‐5p in Tumorigenesis; A Systematic Review.” Pathology—Research and Practice 249, no. March: 154576. 10.1016/j.prp.2023.154576. 37562284

[jex270173-bib-0019] Erceg, S. , J. Munjas , M. Sopić , et al. 2025. “Expression Analysis of Circulating miR‐21, miR‐34a and miR‐122 and Redox Status Markers in Metabolic Dysfunction‐Associated Steatotic Liver Disease Patients With and Without Type 2 Diabetes.” International Journal of Molecular Sciences 26, no. 6: 2392. 10.3390/ijms26062392. 40141039 PMC11942408

[jex270173-bib-0020] Ezaz, G. , H. D. Trivedi , M. A. Connelly , et al. 2020. “Differential Associations of Circulating MicroRNAs With Pathogenic Factors in NAFLD.” Hepatology Communications 4, no. 5: 670–680. 10.1002/hep4.1501. 32363318 PMC7193128

[jex270173-bib-0021] Fornari, F. , L. Gramantieri , C. Giovannini , et al. 2009. “MiR‐122/Cyclin G1 Interaction Modulates p53 Activity and Affects Doxorubicin Sensitivity of Human Hepatocarcinoma Cells.” Cancer Research 69, no. 14: 5761–5767. 10.1158/0008-5472.CAN-08-4797. 19584283

[jex270173-bib-0022] Garcia, N. A. , M. Mellergaard , H. Gonzalez‐king , C. Salomon , and A. Handberg . 2023. “Comprehensive Strategy for Identifying Extracellular Vesicle Surface Proteins as Biomarkers for Non‐Alcoholic Fatty Liver Disease.” International Journal of Molecular Sciences 24, no. 17: 13326. 10.3390/ijms241713326. 37686134 PMC10487973

[jex270173-bib-0023] Hochreuter, M. Y. , M. Dall , J. T. Treebak , and R. Barrès . 2022. “MicroRNAs in Non‐Alcoholic Fatty Liver Disease: Progress and Perspectives.” Molecular Metabolism 65, no. August: 101581. 10.1016/j.molmet.2022.101581. 36028120 PMC9464960

[jex270173-bib-0024] Holcar, M. , J. Ferdin , S. Sitar , et al. 2020. “Enrichment of Plasma Extracellular Vesicles for Reliable Quantification of Their Size and Concentration for Biomarker Discovery.” Scientific Reports 10, no. 1: 1–13. 10.1038/s41598-020-78422-y. 33288809 PMC7721811

[jex270173-bib-0025] Huang, J.‐Y. , K. Zhang , D.‐Q. Chen , et al. 2015. “MicroRNA‐451: Epithelial‐Mesenchymal Transition Inhibitor and Prognostic Biomarker of Hepatocelluar Carcinoma.” Oncotarget 6, no. 21: 18613–18630. 10.18632/oncotarget.4317. 26164082 PMC4621914

[jex270173-bib-0026] Jiang, W. , Y. Xu , J. C. Chen , et al. 2023. “Role of Extracellular Vesicles in Nonalcoholic Fatty Liver Disease.” Frontiers in Endocrinology (Lausanne) 14, no. July: 1–13. 10.3389/fendo.2023.1196831. PMC10392952 37534206

[jex270173-bib-0027] Johnsen, K. B. , J. M. Gudbergsson , T. L. Andresen , and J. B. Simonsen . 2019. “What Is the Blood Concentration of Extracellular Vesicles? Implications for the Use of Extracellular Vesicles as Blood‐Borne Biomarkers of Cancer.” Biochimica et Biophysica Acta (BBA)—Reviews on Cancer 1871, no. 1: 109–116. 10.1016/j.bbcan.2018.11.006. 30528756

[jex270173-bib-0028] Li, Y. , P. Yang , J. Ye , Q. Xu , J. Wu , and Y. Wang . 2024. “Updated Mechanisms of MASLD Pathogenesis.” Lipids in Health and Disease 23, no. 1: 1–15. 10.1186/s12944-024-02108-x. 38649999 PMC11034170

[jex270173-bib-0029] Long, J. K. , W. Dai , Y. W. Zheng , and S. P. Zhao . 2019. “MiR‐122 Promotes Hepatic Lipogenesis via Inhibiting the LKB1/AMPK Pathway by Targeting Sirt1 in Non‐Alcoholic Fatty Liver Disease.” Molecular Medicine 25, no. 1: 1–13. 10.1186/s10020-019-0085-2. 31195981 PMC6567918

[jex270173-bib-0030] Lou, C. , H. Jiang , Z. Lin , et al. 2023. “MiR‐146b‐5p Enriched Bioinspired Exosomes Derived From fucoidan‐Directed Induction Mesenchymal Stem Cells Protect Chondrocytes in Osteoarthritis by Targeting TRAF6.” Journal of Nanobiotechnology 21, no. 1: 1–21. 10.1186/s12951-023-02264-9. 38105181 PMC10726686

[jex270173-bib-0031] Ma, X. , H. Liu , J. Zhu , et al. 2022. “miR‐185‐5p Regulates Inflammation and Phagocytosis Through CDC42 /JNK Pathway in Macrophages.” Genes 13, no. 3: 468. 10.3390/genes13030468. 35328023 PMC8955717

[jex270173-bib-0032] Ellegaard Nielsen, J. , K. Sofie Pedersen , K. Vestergård , et al. 2020. “Novel Blood‐Derived Extracellular Vesicle‐Based Biomarkers in Alzheimer's Disease Identified by Proximity Extension Assay.” Biomedicines 8, no. 7: 199. 10.3390/biomedicines8070199. 32645971 PMC7400538

[jex270173-bib-0033] Mellergaard, M. , A. Askeland , R. W. Rasmussen , et al. 2026. “Circulating Liver‐Derived and CD36^+^ Extracellular Vesicles Correlate With Ectopic Liver Fat and Markers of Fibrosis in Metabolic Dysfunction-Associated Steatotic Liver Disease and Decrease During Weight Loss Intervention.” Journal of Extracellular Vesicles 15, no. 3: e70257. 10.1002/jev2.70257. 41841173 PMC13097540

[jex270173-bib-0034] Nagpal, N. , and R. Kulshreshtha . 2014. “miR‐191: An Emerging Player in Disease Biology.” Frontiers in Genetics 5, no. APR: 1–10. 10.3389/fgene.2014.00099. 24795757 PMC4005961

[jex270173-bib-0035] Nakao, K. , H. Miyaaki , and T. Ichikawa . 2014. “Antitumor Function of microRNA‐122 Against Hepatocellular Carcinoma.” Journal of Gastroenterology 49, no. 4: 589–593. 10.1007/s00535-014-0932-4. 24531873

[jex270173-bib-0036] Nassir, F. , and J. A. Ibdah . 2016. “Sirtuins and Nonalcoholic Fatty Liver Disease.” World Journal of Gastroenterology 22, no. 46: 10084. 10.3748/wjg.v22.i46.10084. 28028356 PMC5155167

[jex270173-bib-0037] Palma, C. , M. K. Masud , D. Guanzon , et al. 2025. “Rapid and High‐Sensitivity Screening of Pregnancy Complications by Profiling Circulating Placental Extracellular Vesicles.” Science Advances 11, no. 9: 1–15. 10.1126/sciadv.adr4074. PMC11864196 40009685

[jex270173-bib-0038] Pirola, C. J. , and T. F. Gianotti . 2015. “Circulating microRNA Signature in Non‐Alcoholic Fatty Liver Disease: From Serum Non‐Coding RNAs to Liver Histology and Disease Pathogenesis.” Gut 64, no. 5: 800–812. 10.1136/gutjnl-2014-306996. 24973316 PMC4277726

[jex270173-bib-0039] Quintás, G. , F. Caiment , I. Rienda , et al. 2022. “Quantitative Prediction of Steatosis in Patients With Non‐Alcoholic Fatty Liver by Means of Hepatic MicroRNAs Present in Serum and Correlating With Hepatic Fat.” International Journal of Molecular Sciences 23, no. 16: 9298. 10.3390/ijms23169298. 36012565 PMC9408888

[jex270173-bib-0040] Rinella, M. E. , J. V. Lazarus , V. Ratziu , et al. 2023. “A Multisociety Delphi Consensus Statement on New Fatty Liver Disease Nomenclature.” Journal of Hepatology 79, no. 6: 1542–1556. 10.1016/j.jhep.2023.06.003. 37364790

[jex270173-bib-0041] Ruoquan, Y. , N. Wanpin , X. Qiangsheng , T. Guodong , and H. Feizhou . 2014. “Correlation Between Plasma miR‐122 Expression and Liver Injury Induced by Hepatectomy.” Journal of International Medical Research 42, no. 1: 77–84. 10.1177/0300060513499093. 24287929

[jex270173-bib-0042] Simonsen, J. B. 2017. What Are We Looking At? Extracellular Vesicles, Lipoproteins, or Both? 920‐922. 10.1161/CIRCRES. 28963190

[jex270173-bib-0043] Szabo, G. , and S. Bala . 2013. MicroRNAs in Liver Disease. 70, no. 4: 646–656. 10.1038/nrgastro.2013.87.MicroRNAs. PMC4091636 23689081

[jex270173-bib-0044] Tan, W. , G. Wang , G. Liu , et al. 2022. “THe Elevation of Mir‐185‐5p Alleviates High‐Fat Diet‐Induced Atherosclerosis and Lipid Accumulation In Vivo and In Vitro via Srebp2 Activation.” Aging 14, no. 4: 1729–1742. 10.18632/aging.203896. 35172278 PMC8908921

[jex270173-bib-0045] Tobaruela‐Resola, A. L. , F. I. Milagro , M. Elorz , et al. 2024. “Circulating miR‐122‐5p, miR‐151a‐3p, miR‐126‐5p and miR‐21‐5p as Potential Predictive Biomarkers for Metabolic Dysfunction‐Associated Steatotic Liver Disease Assessment.” Journal of Physiology and Biochemistry 81: 12751288. 10.1007/s13105-024-01037-8. PMC12738678 39138826

[jex270173-bib-0046] Tobaruela‐Resola, A. L. , F. I. Milagro , P. Mogna‐Pelaez , M. J. Moreno‐Aliaga , I. Abete , and M. Á. Zulet . 2025. “The Use of Circulating miRNAs for the Diagnosis, Prognosis, and Personalized Treatment of MASLD.” Journal of Physiology and Biochemistry 81: 589–609. 10.1007/s13105-025-01110-w. 40668532 PMC12373555

[jex270173-bib-0047] Wang, F. , X. Qi , Z. Li , S. Jin , Y. Xie , and H. Zhong . 2019. “lncRNA CADM1‐AS1 Inhibits Cell‐Cycle Progression and Invasion via PTEN/AKT/GSK‐3&Beta; Axis in Hepatocellular Carcinoma]]>.” Cancer Management and Research 11: 3813–3828. 10.2147/CMAR.S197673. 31118799 PMC6503201

[jex270173-bib-0048] Wang, X. C. , X. R. Zhan , X. Y. Li , J. J. Yu , and X. M. Liu . 2014. “MicroRNA‐185 Regulates Expression of Lipid Metabolism Genes and Improves Insulin Sensitivity in Mice With Non‐Alcoholic Fatty Liver Disease.” World Journal of Gastroenterology 20, no. 47: 17914–17923. 10.3748/wjg.v20.i47.17914. 25548489 PMC4273141

[jex270173-bib-0049] Welsh, J. A. , D. C. I. Goberdhan , L. O'Driscoll , et al. 2024. “Minimal Information for Studies of Extracellular Vesicles (MISEV2023): From Basic to Advanced Approaches.” Journal of Extracellular Vesicles 13, no. 2: e12404. 10.1002/jev2.12404. 38326288 PMC10850029

[jex270173-bib-0050] Wu, C. C. , K. A. Tsantilas , J. Park , et al. 2024. “Mag‐Net: Rapid Enrichment of Membrane‐Bound Particles Enables High Coverage Quantitative Analysis of the Plasma Proteome.” bioRxiv: the preprint server for biology 1–44. 10.1101/2023.06.10.544439.

[jex270173-bib-0051] Wu, X. , C. Yuan , J. Pan , et al. 2024. “CXCL9, IL2RB, and SPP1, Potential Diagnostic Biomarkers in the Co‐Morbidity Pattern of Atherosclerosis and Non‐Alcoholic Steatohepatitis.” Scientific Reports 14, no. 1: 1–20. 10.1038/s41598-024-66287-4. 39013959 PMC11252365

[jex270173-bib-0052] Yamada, H. , K. Suzuki , N. Ichino , et al. 2013. “Associations Between Circulating microRNAs (miR‐21, miR‐34a, miR‐122 and miR‐451) and Non‐Alcoholic Fatty Liver.” Clinica Chimica Acta 424: 99–103. 10.1016/j.cca.2013.05.021. 23727030

[jex270173-bib-0053] Ye, G. , Y. Qin , S. Wang , et al. 2019. “Lamc1 Promotes the Warburg Effect in Hepatocellular Carcinoma Cells by Regulating PKM2 Expression Through AKT Pathway.” Cancer Biology & Therapy 20, no. 5: 711–719. 10.1080/15384047.2018.1564558. 30755064 PMC6605989

[jex270173-bib-0054] Younossi, Z. M. , M. Kalligeros , and L. Henry . 2024. “Epidemiology of metabolic dysfunction-associated steatotic liver disease.” Clinical and molecular hepatology 31: S32–S50. 10.3350/cmh.2024.0431. 39159948 PMC11925440

[jex270173-bib-0055] Zhang, Y. , D. Xiang , X. Hu , Q. Ruan , L. Wang , and Z. Bao . 2020. “Identification and Study of Differentially Expressed miRNAs in Aged NAFLD Rats Based on High‐Throughput Sequencing.” Annals of Hepatology 19, no. 3: 302–312. 10.1016/j.aohep.2019.12.003. 31899128

[jex270173-bib-0056] Zhang, Z. , C. Chen , G. E. Wang , et al. 2011. “Aberrant Expression of the p53‐Inducible Antiproliferative Gene BTG2 in Hepatocellular Carcinoma Is Associated With Overexpression of the Cell Cycle‐Related Proteins.” Cell Biochemistry and Biophysics 61, no. 1: 83–91. 10.1007/s12013-011-9164-x. 21327578

