# Supplementary material for: Extracellular Vesicle‐Based miRNA Profiling in Metabolic Dysfunction‐Associated Steatotic Liver Disease: A Secondary Analysis of the MULTISITE Clinical Trial
Source: J Extracell Biol. 2026 Aug 18;5(8):e70173. doi: 10.1002/jex2.70173 (PMC13487377; doi:10.1002/jex2.70173)
Supplement: Supplementary file 1 [file JEX2-5-e70173-s001.docx]

# **Supplementary material - detailed methods section**

**Extracellular vesicle-based miRNA profiling in metabolic dysfunction-associated steatotic liver disease: a secondary analysis of the MULTISITE clinical trial.**

*Katja Lund Cliff ^a, b^, Dominic Guanzon ^c, d^, Andrew Lai ^c, d^, Katherin Scholz-Romero ^c, d^, Gunna Christiansen ^e^, Aase Handberg ^a, b^, Carlos Salomon ^c^, Maiken Mellergaard ^a, b^.*

**S1 Western blot analysis**

The protein concentration was measured in the five EV enrichment MULTISITE plasma pools and a plasma control sample (Fig. S1) using Pierce ™ BSA Protein Assay kit. (ref:23225). Firstly, the working solution was prepared as 1:50 reagent B, reagent A, whereafter Albumin (BSA) standards were prepared following the manufacturer’s instructions reaching a final BSA concentration of 2000, 1500, 1000, 750, 500, 250, 25, and 0 µg/mL. Following the microplate procedure 20µl of standard were added to a 96 well plate in triplicates, whereafter 10 µl sample + 10 µl Radioimmunoprecipitation assay buffer (RIPA) was added to the plate in duplicates. Lastly, 200 µl working solution was added to all samples and standards, the plate was shaken for 10 s (Microplate BMG, Labtech), covered in aluminum foil, and incubated for 30 min at 37^o^C. The absorbance was measured at 562 nm on a plate reader (TECAN, SPARK 10M). The total protein concentration was calculated using the standard curve, and the amount (µl) needed from each sample to collect 30 µg of protein for western blot analysis. Whereas the plasma control sample was diluted 200 times, and only 0.15 µg of protein was used (Fig. S1+S2).

The protein was diluted in RIPA to reach a total volume of 25 µl, from which 20 µl was mixed with 10.5 µl Loading buffer. Hereafter, 25 µl was added to a Bolt™ 4-12 % Bis-Tris Plus WedgeWell™ Gell (Invitrogen, ref: NW04120BOX) and run at 200 V for 25min. The gel was transferred to a Wet Immobilon-FL PVDF Membrane (Millipore®, Ref: IPFL00010) using a Mini-PROTEAN Tetra system at 100 V for 60min. Hereafter, the membrane was washed using TBS-TWEEN® (Sigma, #P1379-500mL) and incubated in the dark on a shaker for 60 min in 15 mL TBS Blocking Buffer (Intercept®, Albumin, #927-60001). The protein recovery of EV related proteins was determined using the primary antibodies CD63 rabbit (Cell signaling, #52090, Molecular Weight (MW)~25-60kDa, DF 1:500), Flotillin Rabbit (Cell signaling, #18634T, MW~49kDa, 1:1000), and lipoprotein co-isolation was checked using Anti-Apolipoprotein A 1 Rabbit (Apo-A1) (Abcam, #AB52945, MW~28kDa, DF1:1000). After an overnight incubation at 4^o^C with the primary antibodies, the membranes were washed with washing buffer (20X TBS in diH20, with 1 % TWEEN-20) and incubated with a Goat Anti-Rabbit secondary antibody (DF1:10.000) (LI-COR®, #925-32211). The membranes were washed three times and measured using a ChemiDoc™ MP Imaging system (BIO-RAD).

**S2 Next generation sequencing (NGS)**

**S2.1 RNA extraction and quantification**

RNA extraction was performed using the Nordic Biotek Exosomal RNA extraction kit for plasma/serum (cat: 58000, 50 prep) following the manufactures instructions. Lysis Buffer A was warm to 60^o^C for 20 min prior to analysis. Wash Solution A was diluted with 42 µl of 96-100 % ethanol as instructed by the manufacturer. The 148 EV enriched samples underwent RNA-Extraction in five batches over a duration of three days. On the day of analysis one batch of samples were thawed at room temperature and the 100 µl of EV enriched samples were transported to a DNA LoBind 2 ml Eppendorf tube (lot: L2026031, 250 Safe-Lock tubes). The samples were diluted by adding 100 µl of PBS to reach a total volume of 200 µl for RNA extraction. Next step was to add 300 µl warm Lysis Buffer A and 37.5 µl Lysis Additive B to each sample. Hereafter, all samples were mixed by vortex for 10 sec following a 10 min incubation at room temperature. Before RNA extraction could continue, the beads were removed by placing the samples on a magnetic stand for 2 min, whereafter the supernatant were collected and transferred to a new DNA LoBind 2ml Eppendorf tube. Then add 500 µl 96-100 % ethanol to each sample and mixed by vortex for 10 sec. Transfer 500 µl of the mixture into a Mini Spin Column (cat: 58000) and centrifuge for 1 min at 3,3000g. Discard the flowthrough and reassemble the spin column with its collection tube. This step was repeated by transferring the remaining sample to the spin column. Next step is to wash the samples twice by adding 550 µl Wash Solution A, centrifuge for 30 sec at 3,3000g and discard the flowthrough. After the last wash, spin the column empty for 1 min at 13,000g. Discard the collection tube and transfer the spin column to a fresh 1.7 ml Elution tube (cat:58000). Add 30µl of Elution Solution A to the column and centrifuge for 1 min at 400g, following a 2 min at 5,8000g. Transfer the eluted buffer back into the column and centrifuge again for 1 min at 400g, following 2 min at 5,8000g. EV RNA is now ready for downstream application.

Immediately after RNA extraction each batch of samples went through RNA quantification using Qubit (Invitrogen, Qubit microRNA assay kit, 500 assays, lot: 2507896, ref: Q32881) following the manufactures instructions. Qubit ™ working solution were prepared by diluting Qubit ™ microRNA reagent 1:200 in Qubit ™ microRNA buffer. Qubit consisting of two standards, a low at 0. ng/µl and high at 10 ng/µl which is prepared by mixing 10 µl of standard with 190 µl of the prepared working solution. Each RNA sample was prepared by mixing 4 µl of RNA with 196 µl working solution. All samples were gently mixed by vortex for 1 sec and incubated for 2-3 min at room temperature. Lastly, all samples were measured using the Qubit instrument (Qubit 4 Fluorometer, Invitrogen, Thermo Fisher Scientific, software version: APP 2.01 + MCU v0.26).

**S2.2 miRNA Library prep**

**S2.2.1 NEXTFLEX® 3`- and 5´ adaptor ligations**

MiRNA library prep was performed using NEXTFLEX, small RNA-Seq Kit v4 with Unique Dual Indices (UDIs) (PerkinElmer, V4, Cat # NOVA-5132-42 and #NOVA-5132-43). First step was to prepare a 3`Adenylted Adaptor ligation master mix containing 1µl 3´Adenylated adaptor (1/4 DF), 12.5 µl 3´Ligation buffer, and 1.5 µl ´3 Ligation enzyme mix for each sample. 15 µl master mix was added to a 0.2 ml non-skirted 96 well PCR plate (Thermo Scientific AB-0600). The RNA samples were concentrated using a (Concentrator-Plus, Eppendorf), whereafter 5 µl RNA was added to the 96 wells plate, reaching a total volume of 20 µl. Flat 8 cap strips (thermos Scientific, AB-0784) were used to seal the plate. Hereafter, the plate was vortexed, mixed at 1900 RPM for 1 min using a Thermo-Mixer, vortexed again, and lastly spined with 1000g for 1 min (Avanti-J-15R centrifuge, Beckman Coulter). The plate was incubated for 60 min at 25^o^C and 5 min at 4^o^C using a thermal cycler (T100 ™, BIO-RAD). Hereafter, 4 µl of adaptor inactivation mix was added and the plate was mixed as previously described. The plate was incubated for 2 min at 70^o^C and 5 min at 4^o^C. Next step was to prepare a 5´adaptor ligation master mix containing 1 µl 5´adaptor (1/4 DF), 3 µl 5´ Ligation buffer, and 2 µl 5´Ligation enzyme mix for each sample. 6 µl of master mix was added to the plate and mixed as previously described. The plate was incubated for 60 min at 20^o^C and 5 min at 4^o^C.

**S2.2.2 Reverse transcription-first strand synthesis and beads cleanup**

A reverse transcription-first strand master mix was prepared containing 1 µl RT primer, 7 µl RT buffer, and 2 µl RT enzyme mix for each sample. 10 µl of master mix was added to the plate and mixed as previously described. The plate was incubated for 60 min at 50^o^C, 5 min at 90^o^C, and 5 min at 4^o^C. Beads cleanup was performed by adding 40 µl adaptor depletion solution, 40 µl Cleanup beads, and 90 µl isopropanol to each well and mixed by pipetting. The plate was incubated for 5min at room temperature following a magnetization for 5 min. Hereafter, two washing steps were performed using 200 µl 80 % ethanol. After all residual liquid was removed, the beads were resuspended in 18 µl Nuclease-free water and incubated for 2 min. Lastly, the plate was magnetized for 3 min and 1 6µl of supernatant was transferred to a new 96 well plate.

**S2.3 PCR amplification**

For PCR amplification 4 µl UDI barcoded primer mix (A different barcode for each sample) and 6 µl small RNA PCR master mix was added to the plate. The plate was incubated for 30 sec at 98^o^C following 23 cycles of 10 sec at 98^o^C, 20 sec at 65^o^C, 15 sec at 72^o^C, and lastly 2 min at 72^o^C. Stopping point. The plate was stored at -20^o^C for two days until all samples were ready for gel size selection.

**S2.4 Gel size selection and cleanup**

Each PCR product was stained with 5.2 µl 6X gel loading dye (purple, no SDS, B70255, New England Biolabs, Lot #10208667) and all sample was loaded onto a 6 % TBE-PAGE gel (ref: EC6265BOX, Lot: 25031744, Invitrogen) on a Mini gel Tank (Invitrogen). In an adjacent lane 10 µl Low molecular weight (MW) ladder (Quick-Load purple DNA ladder, cat #NO557S, New England Biolabs) was loaded as a control guide. The gel was run with 200V for 30 min (PowerPac ™ Basic, Bio-Rad) whereafter the gel was stained and incubated for 10 min on a shaker (gently) with 5 µl SYBR Gold (Nucleic acid stain, lot: 2700997, Invitrogen) diluted in 50 ml TBE 1X (TBE running Buffer 5X, lot: 3123486, Novex by Life technologies). The samples were visualized using a UV transilluminator and the gel was cut between 150-200 bp using a clean razor. Each sample gel piece was then added to their respective gel crusher tube in a 2ml round bottom tube and centrifuged for 2 min at 20.000g. The gel crusher tube was discarded and 300 µl IDTE pH 8 (1X TE solution, lot #903137, nuclease free, Integrated DNA technologies) was added to each sample and soaked overnight with 70 RPM agitation (Vortemp 56, Labnet, temp off) at room temperature. The following day all samples were pulse spined, transferred to a Spin-X centrifuge tube, centrifuged for 2 min at 16.000g whereafter the Spin filter was discarded. For beads cleanup 50 µl NEXTFLEX cleanup beads (lot #R2147617) and 350 µl isopropanol was added to each sample and incubated for 10 min at room temperature. The samples were magnetized to remove the supernatant, whereafter the beads were washed twice using 950µl 80 % ethanol. The beads were dried for 3 min and resuspended in 13 µl resuspension buffer (lot #R214714). After 2 min incubation and 3 min magnetizing 12 µl supernatant (sequencing library) were collected and transferred to a new 96 well PCR plate. The library PCR plates were stored at -20^o^C overnight.

**S2.5 cDNA quantification**

cDNA quantification was performed using the KAPA Library Quant Kit (Illumina, Universal qPCR mix, ref: 7960140001, lot: 515766, KAPA Biosystems). The prepared library was thawed on ice, vortex for 20 sec, mixed for 1 min at 1900 RPM and spined down for 1 min at 1000g. Hereafter, a dilution DF10.000 and DF50.000 was created using distilled water (UltraPure ™ DNAse & RNAse Free, ref:10977-015, lot:2922571, Invitrogen) for the first 20 samples in a 0.2 ml non-skirted 96-well PCR plate. 4 µl of each dilution from each sample was added to a MicroAmp® Fast Optimal 96-well reaction Plate with barcode (0.1mL, ref: 4346906, Applied Biosystems) in duplicates (80 wells in total). 4µl of 6 DNA standards, 20pM, 2pM, 0.2pM, 0.02pM, 0.002pM and 0.0002pM, and a distilled water control was added to the MicroAmp plate in duplicates. A qPCR master mix was prepared as described by the manufacturers and 6 µl was added to each well in the 96 well plate. The plate was sealed using a MicroAmp® Optimal Adhesive Film (ref: 4311971, lot:33NM6Y, Applied Biosystems), vortexed for 20 sec, mixed for 1 min at 1900 RPM, and spun down for 1 min at 1000g. The plate was transferred to a quantitative reverse-transcription polymerase chain reaction (qRT-PCR) instrument (QuantStudio 3, Applied Biosystems) which underwent the following cycles: 5 min at 95^o^C (1 cycle), 30 sec at 95^o^C and 45 sec at 60^o^C (35 cycles) and analyzed using the QuantStudio™ Design & Analysis Software v1.5.3. The concentration of cDNA in each sample was calculated using the Ct value and standard curve provided by the qPCR analysis. Lastly, all samples were diluted to reach the same concentration and pooled in two pools.

**S3. Statistics - Biomarker analysis**

In this study we used the PyCaret's classification module to train fourteen different algorithms for the two outcomes as previously described. PyCaret (version 3.3.2), is an automated machine learning low-code library in Python that automates the machine learning workflow. For all models Python (version 3.11.0) were used. The fourteen machine learning algorithms (Random Forest (RF), SVM-Radial Kernel (RBFSVM), Extra Trees Classifier (ET), Gaussian Process Classifier (GPC), Decision Tree Classifier (DT), Multi-layer Perceptron (MLP), Light Gradient Boosting Machine(LIGHTBM), K Neighbors Classifier (KNN), Gradient Boosting Classifier (GBC), Ada Boost Classifier (ADA), Logistic Regression (LR), Linear Discriminant Analysis (LDA), Quadratic Discriminant Analysis (QDA) and Naive Bayes(NB)), were trained and evaluated first on tenfold cross-validation, then on the test data. The best model selection was selected on accuracy, which is calculated based on the total correct predictions divided by the total number of predictions. Additionally, the optimal sensitivity, specificity, positive predictive value, negative predictive value, area under the receiver operating characteristic curve, area under precision recall graph and Brier score were calculated for the training and test data. Finally, feature importance was calculated for the best model, providing an overall assessment of which biomarkers are most crucial in distinguishing between control and case samples.

**S3.1 Generative Artificial Intelligence-based workflows**

To explore whether synthetic data could enhance model robustness and reduce overfitting, we implemented a Generative Artificial Intelligence (GAI) approach for synthetic data augmentation. Control samples were used as the basis for generating two synthetic datasets containing 1,000 and 10,000 artificial individuals, respectively. Synthetic data generation was performed using the SingleTableMetadata module from the Synthetic Data Vault (SDV) library, which provides tools for modelling and reproducing complex tabular datasets. A Gaussian Copula Synthesizer was selected for this task, as it captures multivariate dependencies by modelling correlations between features through a copula-based framework. The synthesizer was trained on the original control dataset to learn the joint distribution and structural relationships among miRNAs. Once the model parameters converged, synthetic miRNA expression profiles were sampled from the trained model. The fidelity and utility of the synthetic datasets were evaluated using TableEvaluator, which assesses similarity to real data across distributional, correlation, and machine learning efficacy metrics. Only synthetic datasets that maintained adequate statistical alignment with the real data were incorporated into subsequent analyses. Each validated synthetic dataset was pooled with the original real-world training samples to generate augmented training datasets. Random forest classifiers were then trained on these augmented datasets using identical hyperparameter settings as in the baseline models to ensure comparability. This GAI-based augmentation allowed us to test whether increasing sample diversity and size could improve classification performance or model generalisability.
