# Supplementary material for: Extracellular Vesicle‐Based miRNA Profiling in Metabolic Dysfunction‐Associated Steatotic Liver Disease: A Secondary Analysis of the MULTISITE Clinical Trial
Source: J Extracell Biol. 2026 Aug 18;5(8):e70173. doi: 10.1002/jex2.70173 (PMC13487377; doi:10.1002/jex2.70173)
Supplement: Supplementary file 2 [file JEX2-5-e70173-s002.docx]

**Supplementary figures and tables**

**Extracellular vesicle-based miRNA profiling in metabolic dysfunction-associated steatotic liver disease: a secondary analysis of the MULTISITE clinical trial.**

*Katja Lund Cliff ^a, b^, Dominic Guanzon ^c, d^, Andrew Lai ^c, d^, Katherin Scholz-Romero ^c, d^, Gunna Christiansen ^e^, Aase Handberg ^a, b^, Carlos Salomon ^c^, Maiken Mellergaard ^a, b^.*

The supporting information can be downloaded at:

**Figure S1: Protein quantification of isolated EV pools for EV validation**

Fig. S1. Protein quantification using Pierce™ BCA Protein Assay for 5 isolated EV pools and a plasma control sample for Western blot analysis. **A)** Standard curve, and **B)** Protein quantification for Pool1 (Lean control group), Pool2 (Obesity control group), Pool3 (MASLD group at baseline), Pool4 (MASLD group after 1 month), Pool5 (MASLD group after 5 months), and Plasma control.

**Figure S2: Western blot analysis of APO-A1**

Fig. S2: **A)** Western blot analysis of APO-A1 contamination in 5 isolated EV pools (1. Lean control, 2. Obesity control, 3. MASLD baseline, 4. MASLD 1-month, 5. MASLD 5-months), and a plasma control, PBS control, and a loading buffer (LB) control. For each of the 5 plasma pools, 30µl of proteins were added, whereas the plasma control was diluted 200 times, and only 0.15µg was added. **B)** The intensity of each band was calculated using ImageJ software, with correction for the dilution factor of 200 for the plasma control.

**Figure S3: Real-Time qPCR cDNA quantification - Standard curve (example 1 of 8)**

Fig. S3: cDNA quantification using RT-qPCR illustrated with **A)** the standard curve (example 1 of 8), and **B)** the cDNA quantification for al individual samples divided in the five study groups.

**Figure S4: Heatmap – MASLD baseline vs Obese control group**

B. 4 significantly differentially expressed miRNAs.

C. Top 20 differentially expressed miRNAs

Fig. S4: **A)** Heatmap showing 4 significantly differentially expressed miRNAs and **B)** top 20 differentially expressed miRNAs between the MASLD and obesity control groups at baseline. miRNAs with a p-value<0.05 and log2foldchange≥0.29 (equivalent to FC≥1.22) are considered significantly differentially expressed.

**Figure S5: Volcano plot for the MASLD intervention group**

Fig. S5: Volcano plot illustrating differential expressed miRNAs between **A)** MASLD after 5 months vs MASLD at baseline, **B)** MASLD after 1 month vs MASLD at baseline, **C)** MASLD after 5 months vs MASLD after 1 month. miRNAs with a p-values<0.05 and log2foldchange≥0.29 (equivalent to FC≥1.22) are considered significantly differentially expressed.

**Figure S6: Weight loss intervention: Liver fat content (PDFF) and BMI**

Fig. S6: Weight loss intervention analysed using a Wilcoxon signed rank test for the MASLD group (n=28) at Baseline (B), 1 month (1m), and 5 months (5m) for **A)** liver fat content (PDFF, %) and **B)** BMI (kg/m^2^). Data presented as median, interquartile range, and significant level: P<0.05 (*), P<0.01 (**), p<0.001 (***), p<0.0001 (****).

**Figure S7: Classification performance and synthetic data argumentation of EV-derived miRNA panel 1 and 2.**

**Fig. S7. A)** A leave-one-out cross-validation model with a ROC, Precision-recall, and Calibration curve was used to assess the diagnostic performance of individual and combined miRNAs in panel 1 (miR-10a, miR-122, miR-146b, and miR-185) for distinguishing MASLD from the obesity control group, and **B)** panel 1 and **C)** panel 2 (miR-10a, miR-122, miR-146b, miR-185, miR-331, miR-499a, and miR-6773) for identifying high liver fat among all individuals with obesity. **D)** ROC curves showing synthetic samples generated using generative AI of miRNA panel 1 for distinguishing MASLD from the obesity control group, blue (non), red (synthetic 1000), and green (synthetic 10000), and **E)** for identifying high liver fat among all individuals with obesity, blue (non), red (synthetic 1000), and green (synthetic 10000). **F)** Volcano plot illustrating differential expressed miRNAs (Panel 2) between the high liver fat group (n=18) and low liver fat group (n=18). miRNAs with a p-value<0.05 and log2foldchange≥0.29 (equivalent to FC≥1.22) are considered significantly differentially expressed. **G)** ROC curves showing synthetic data argumentation of miRNA panel in identifying high liver fat among all individuals with obesity. Blue (non), red (synthetic 1000), and green (synthetic 10000).

Sup. tables

**Table S1: Database search – related diseases**

| **MASLD Baseline vs Obesity control group** | | | |
| --- | --- | --- | --- |
| **miRNA** | **Regulated** | **GeneCaRNA.com** | **miRBase** |
| hsa-miR-122-5p | Up | MASLD & Hepatitis C | Hepatocellular carcinoma |
| hsa-miR-146b-5p | Up | Glioma & Breast Cancer | Glioma & Breast cancer |
| hsa-miR-185-5p | Up | Lung Non-Small Cell Carcinoma & Kidney Cancer | Hepatocellular carcinoma & Cardiac hypertrophy |
| hsa-miR-10a-5p | Down | Leukaemia & Chronic Myeloid | Colorectal & Pancreatic cancer |
| **MASLD 5 months vs MASLD Baseline** | | | |
| hsa-miR-191-5p | Down | Hepatocellular carcinoma | Colorectal & Lung cancer |
| hsa-miR-26a-5p | Down | MASLD & Colorectal cancer & Hepatocellular carcinoma | Hepatocellular Carcinoma & Acute myeloid leukaemia |
| hsa-miR-146a-5p | Down | Psoriatic Arthritis & Alzheimer´s Disease | Breast cancer & Hepatocellular Carcinoma |
| hsa-miR-151a-5p | Down | MASLD & Diffuse Large BlCell Lymphoma | NAFLD & Hepatocellular carcinoma |
| hsa-miR-335-5p | Down | Breast Cancer & Leiomyoma | Acute lymphoblastic leukaemia & Multiple myeloma |
| hsa-miR-30d-5p | Down | Medulloblastoma & Lung Non-Small Cell Carcinoma | NAFLD & Chronic lymphocytic leukaemia |
| hsa-miR-98-5p | Down | Niemann-Pick Disease & Squamous Cell Carcinoma | Breast cancer & Lung cancer |
| hsa-miR-584-5p | Down | Renal Cell Carcinoma  & Mesothelioma & Malignant. | Hepatocellular carcinoma & Carcinoma, Non-Small-Cell Lung |
| hsa-miR-451a | Up | Glioblastoma & Gastric Cancer | Breast Neoplasms & Hepatocellular carcinoma & Diabetes |
| hsa-miR-125b-5p | Up | Pancreatitis & Glioblastoma | Prostate cancer |
| hsa-miR-1260a | Up | Diabetes Mellitus | Breast Neoplasms |

Table S1: The miRbase.org and GeneCaRNA.com database search. Related diseases for 14 selected Up/Downregulated miRNAs.

**Table S2: Correlations analysis – Clinical parameters**

|  | **miR-122** | **miR-10a** | **miR-146b** | **miR-185** |
| --- | --- | --- | --- | --- |
| **Liver fat (PDFF, %)** | R^2^ = 0.19 (**) | R^2^ = 0.04 | R^2^ = 0.01 | R^2^ = 0.009 |
| **ALT (U/L)** | R^2^ = 0.44 (******) | R^2^ = 0.11 | R^2^ = 0.001 | R^2^ = 0.02 |
| **AST (U/L)** | R^2^ = 0.3 (***) | R^2^ = 0.06 | R^2^ = 0.004 | R^2^ = 0.03 |
| **HDL cholesterol (mmol/L)** | R^2^ = 0.16 (*) | R^2^ = 0.12 (*) | R^2^ = 0.6 | R^2^ = 0.06 |
| **LDL cholesterol (mmol/L)** | R^2^ = 0.01 | R^2^ = 0.001 | R^2^ = 0.03 | R^2^ = 0.001 |
| **GGT (U/L)** | R^2^ = 0.12 (*) | R^2^ = 0.08 | R^2^ = 0.01 | R^2^ = 0.0003 |
| **HOMA-IR** | R^2^ = 0.07 | R^2^ = 0.06 | R^2^ = 0.01 | R^2^ = 0.002 |
| **Triglycerides (mmol/L)** | R^2^ = 0.1 | R^2^ = 0.01 | R^2^ = 0.1 | R^2^ = 0.008 |
| **Insulin (pmol/L)** | R^2^ = 0.06 | R^2^ = 0.06 | R^2^ = 0.01 | R^2^ = 0.0004 |

Table S2: Linear regression analysis between the expression level of selected miRNAs and clinical parameters. Data presented as R^2^ and BH adjusted significant levels: p<0.05 (*), p<0.01 (**), p<0.001 (***), p<0.000001 (******). HOMA-IR: homeostasis model assessment 2 for insulin resistance.

**Table S3: LOOCV performance metrics of EV-Derived miRNA panels for discriminating MASLD and high liver fat content among individuals with obesity**

| **Analysis** | **AUROC** | **Sensitivity (95%, CI)** | **Specificity (95%, CI)** | **AUPRC** | **Brier score** |
| --- | --- | --- | --- | --- | --- |
| **Panel 1 MASLD vs Obesity control group** | | | | | |
| Combination | 0.75 | 75% (46 - 92%) | 74% (37 - 91%) | 0.63 | 0.23 |
| miR-146b | 0.81 | 79% (54 - 96%) | 69% (43 - 89%) | 0.76 | 0.21 |
| miR-185 | 0.71 | 67% (38 - 88%) | 69% (40 - 89%) | 0.59 | 0.28 |
| miR-10a | 0.68 | 62% (8 - 79%) | 86% (31 - 97%) | 0.57 | 0.23 |
| miR-122 | 0.64 | 71% (33 - 88%) | 60% (29 - 80%) | 0.53 | 0.26 |
| **Panel 1 High fat group (Top 30%) among all individuals with obesity** | | | | | |
| Combination | 0.72 | 78% (45 - 94%) | 59% (29 - 76%) | 0.55 | 0.36 |
| miR-146b | 0.56 | 72% (15 - 89%) | 61% (15 - 75%) | 0.31 | 0.28 |
| miR-185 | 0.5 | 89% (50 - 100%) | 20% (0 - 37%) | 0.71 | 0.25 |
| miR-10a | 0.77 | 72% (11 - 89%) | 83% (54 - 95%) | 0.52 | 0.22 |
| miR-122 | 0.71 | 56% (28 - 78%) | 78% (46 - 95%) | 0.54 | 0.22 |
| **Panel 2 High fat group (Top 30%) among all individuals with obesity** | | | | | |
| combination | 0.89 | 100% (72 - 100%) | 63% (49 - 80%) | 0.81 | 0.21 |
| miR-146b | 0.56 | 72% (15 - 89%) | 61% (15 - 76%) | 0.31 | 0.28 |
| miR-185 | 0.5 | 89% (56 - 100%) | 20% (0 - 37%) | 0.71 | 0.25 |
| miR-10a | 0.77 | 72% (17 - 89%) | 83% (49 - 95%) | 0.52 | 0.22 |
| miR-122 | 0.71 | 56% (28 - 78%) | 78% (46 - 95%) | 0.54 | 0.22 |
| miR-499a | 0.76 | 44% (22 - 67%) | 95% (57 - 100%) | 0.69 | 0.17 |
| miR-6773 | 0.64 | 56% (11 - 78%) | 78% (44 - 90%) | 0.4 | 0.25 |
| miR-331 | 0.5 | 61% (32 - 83%) | 46% (5 - 71%) | 0.69 | 0.25 |

Table S3: A leave-one-out cross-validation (LOOCV) model was used to assess the diagnostic performance of individual and combined miRNAs for MASLD baseline vs Obesity control group (Panel 1), and for High liver fat group (Top 30%, n=18) vs all individuals with obesity (n=59) (Panel 1 and 2). Area Under the Receiver Operating Characteristic (AUROC), Area Under the Precision-Recall Curve (AUPRC).

Supplementary material for NAFLD+Mets grouping:

**Table S4: Differential expression analysis of baseline groups:**

| **NAFLD+Mets vs Obesity control group** | | | |
| --- | --- | --- | --- |
| **miRNA** | **P-value** | **Log2FoldChange** | **Regulated** |
| hsa-miR-122-5p | 0.004427 | 0.718841 | Up |
| hsa-miR-10a-5p | 0.028221 | "-0.313566 | Down |
| hsa-miR-146b-5p | 0.0119405 | 0.345967 | Up |
| hsa-miR-4508 | 0.031089 | 2.394752 | Up |

Table S4: Differential expression analysis of NAFLD+Mets vs obese control group. miRNAs with a p-value<0.05 and log2foldchange≥0.29 (equivalent to FC≥1.22) are considered significantly differentially expressed.

**Table S5: Differential expression analysis for NAFLD+Mets intervention group:**

| **NAFLD+Mets Intervention: Downregulated miRNAs** | | | **NAFLD+Mets intervention: Upregulated miRNAs** | | |
| --- | --- | --- | --- | --- | --- |
| NAFLD+Mets 5 months vs NAFLD+Mets baseline | | | NAFLD+Mets 5 months vs NAFLD+Mets Baseline | | |
| miRNA | P-value | Log2FoldChange | miRNA | P-value | Log2FoldChange |
| hsa-miR-335-5p | 0.000099 | -0.680513 | hsa-miR-1260a | 0.002324 | 2.925738 |
| hsa-miR-191-5p | 0.000412 | -0.423317 | hsa-miR-374a-5p | 0.006968 | 3.004307 |
| hsa-miR-584-5p | 0.001025 | -2.763553 | hsa-miR-125b-5p | 0.010135 | 1.102356 |
| hsa-miR-4433b-5p | 0.009141 | -2.321606 | hsa-miR-451a | 0.010955 | 0.509323 |
| hsa-miR-30d-5p | 0.011754 | -0.435848 | hsa-miR-660-5p | 0.013807 | 3.022003 |
| hsa-miR-151a-5p | 0.012651 | -0.503250 |  |  |  |
| hsa-miR-26a-5p | 0.013425 | -0.348520 |  |  |  |
| hsa-miR-98-5p | 0.023486 | -1.265506 |  |  |  |
| hsa-miR-146a-5p | 0.046572 | -0.249783 |  |  |  |
| NAFLD+Mets 1 month vs NAFLD+Mets baseline | | | NAFLD+Mets 1 month vs NAFLD+Mets baseline | | |
| hsa-miR-191-5p | 0.001604 | -0.378782 | hsa-miR-337-5p | 0.003949 | 4.264996 |
| hsa-miR-26a-5p | 0.003317 | -0.415869 | hsa-miR-451a | 0.008545 | 0.526984 |
| hsa-miR-584-5p | 0.004771 | -2.373433 | hsa-miR-99a-5p | 0.023333 | 0.327359 |
| hsa-miR-335-5p | 0.014633 | -0.428818 | hsa-miR-100-5p | 0.024131 | 0.388655 |
| hsa-miR-93-5p | 0.018387 | -1.596799 | hsa-miR-186-5p | 0.039247 | 0.979009 |
| hsa-miR-98-5p | 0.019223 | -1.315872 |  |  |  |
| hsa-let-7e-5p | 0.020004 | -2.151323 |  |  |  |
| hsa-let-7f-5p | 0.029850 | -0.449574 |  |  |  |
| NAFLD+Mets 5 months vs NAFLD+Mets 1 month | | | NAFLD+Mets 5 months vs NAFLD+Mets 1 month | | |
| hsa-miR-30d-5p | 0.000168 | -0.659509 | hsa-miR-1260a | 0.005279 | 2.687453 |
| hsa-miR-4433b-5p | 0.014997 | -2.178636 | hsa-miR-31-5p | 0.004674 | 7.908155 |
|  |  |  | hsa-miR-181a-5p | 0.017290 | 0.756891 |
|  |  |  | hsa-miR-324-5p | 0.040387 | 1.428582 |

Table S5: Differential expression analysis in the NAFLD+Mets group: 5 months vs Baseline, 1 month vs Baseline, and 5 months vs 1 month. Table divided in down-regulated (left) and up-regulated (right) miRNAs. miRNAs with a p-value<0.05 and a log2foldchange>0.29 (equivalent to FC≥1.22) are considered significantly differentially expressed.
